# KDM4B is a Master Regulator of the Estrogen Receptor Signalling Cascade

**DOI:** 10.1093/nar/gkt469

**Published:** 2013-05-30

**Authors:** Luke Gaughan, Jacqueline Stockley, Kelly Coffey, Daniel O’Neill, Dominic L. Jones, Mark Wade, Jamie Wright, Madeleine Moore, Sandy Tse, Lynsey Rogerson, Craig N. Robson

**Affiliations:** ^1^Solid Tumour Target Discovery Group, Northern Institute for Cancer Research, Newcastle University, Paul O’Gorman Building, Newcastle Upon Tyne, NE2 4HH, UK, ^2^Beatson Institute for Cancer Research, Garscube Estate, Switchback Road, Bearsden, Glasgow, G61 1BD, ^3^Blizard Institute of Cell and Molecular Science, Barts and The London School of Medicine and Dentistry, 4, Newark street, London, E1 2AT, UK, ^4^Musculoskeletal Research Group, Institute of Cellular Medicine, 4th Floor Catherine Cookson, Medical School, Framlington Place, Newcastle University, NE2 4HH, UK and ^5^Molecular Pathology Group, Breakthrough Breast Cancer Research Unit, Paterson Institute for Cancer Research, Wilmslow Road, M20 4BX, UK

## Abstract

The importance of the estrogen receptor (ER) in breast cancer (BCa) development makes it a prominent target for therapy. Current treatments, however, have limited effectiveness, and hence the definition of new therapeutic targets is vital. The ER is a member of the nuclear hormone receptor superfamily of transcription factors that requires co-regulator proteins for complete regulation. Emerging evidence has implicated a small number of histone methyltransferase (HMT) and histone demethylase (HDM) enzymes as regulators of ER signalling, including the histone H3 lysine 9 tri-/di-methyl HDM enzyme KDM4B. Two recent independent reports have demonstrated that KDM4B is required for ER-mediated transcription and depletion of the enzyme attenuates BCa growth *in vitro* and *in vivo*. Here we show that KDM4B has an overarching regulatory role in the ER signalling cascade by controlling expression of the *ER* and *FOXA1* genes, two critical components for maintenance of the estrogen-dependent phenotype. KDM4B interacts with the transcription factor GATA-3 in BCa cell lines and directly co-activates GATA-3 activity in reporter-based experiments. Moreover, we reveal that KDM4B recruitment and demethylation of repressive H3K9me3 marks within upstream regulatory regions of the *ER* gene permits binding of GATA-3 to drive receptor expression. Ultimately, our findings confirm the importance of KDM4B within the ER signalling cascade and as a potential therapeutic target for BCa treatment.

## INTRODUCTION

Breast cancer (BCa) is the most prevalent malignancy in women, and almost two-thirds of newly diagnosed BCas express the estrogen receptor (ER), which remains the primary target for disease therapy. By preventing binding of the ER to its activating hormone estrogen, endocrine therapy, via use of tamoxifen, is responsible for decreasing BCa incidence by ∼50% (1). Most malignancies, however, become refractory to treatment and progress as a consequence of ill-defined molecular events that enable ER function in the absence of bound hormone (2).

The ER is a member of the nuclear hormone receptor superfamily of transcription factors that regulate genes involved in breast epithelial cell division and transformation (3). Pioneer factors, such as FOXA1 and GATA-3, facilitate loading of the receptor onto *cis*-acting enhancer elements of target genes and permit ordered recruitment of numerous distinct co-regulator families, which act to regulate the estrogenic transcriptional response (4). Importantly, several lines of evidence suggest imbalance in the relative levels of these co-regulators is involved in the development of BCa. For example, overexpression of the histone acetyltransferase enzyme AIB1 has been reported in 10% of breast tumours (5), while flux to co-repressor proteins SMRT and N-CoR levels has been suggested to be causative for tamoxifen resistance (6,7).

Although our present understanding is limited, emerging evidence implicates an important role for enzymes that regulate reversible histone lysine methylation in ER signalling and BCa (8). Histone lysine methylation is catalysed by the large family of SET domain-containing histone methyltransferase (HMT) enzymes that is reversed by the activity of Jumonji (Jmj) and KDM1 histone demethylase (HDM) enzymes. Discriminate mono-, di- and tri-methylation and respective demethylation of lysines within histones H3 and H4 encompassing euchromatic domains acts as a genome-wide epigenetic switch that can either activate or repress transcription (9,10). For example, tri-methylation of histone H3 lysine 4 (H3K4me3) is generally associated with transcriptionally active loci, while tri-methylation of histone H3 lysine 9 (H3K9me3) is generally associated with transcriptionally silenced, heterochromatic regions. The demonstration that the histone H3 lysine 27 HMT enzyme EZH2 promotes anchorage-independent growth of the H16N2 BCa cell line (11), and that both EZH2 (12) and the HDM KDM1A (13) are overexpressed in BCa and up-regulate the transcriptional activity of the ER (8) suggest a role for aberrant histone methylation in breast malignancy.

More recently, two separate reports have suggested that the H3K9me3/me2-specific demethylase enzyme KDM4B is an ER co-regulator by demonstrating that depletion of the enzyme reduces both receptor-mediated transcription and growth of BCa cells *in vitro* and *in vivo* (14,15). In addition, the finding that *KDM4B* is an ER-responsive gene (16) suggested the existence of a positive-acting regulatory loop between the two proteins in which estrogen-dependent elevation of KDM4B feeds forward to further potentiate ER function and up-regulate the estrogenic response in BCa cells.

Consistent with these findings, from an siRNA library screen, we identified KDM4B as a regulator of the ER signalling cascade and demonstrated that it is recruited to *cis*-regulatory elements of estrogen-dependent genes. Furthermore, KDM4B depletion markedly reduced ER-target gene expression in several BCa cell lines, including MCF-7, T47D and BT-474. Importantly, investigating the role of KDM4B in the receptor signalling cascade further, we show for the first time that the demethylase is required for *ER* gene expression via co-activation of GATA-3, an important regulator of *ER* transcription (17). KDM4B is recruited to both promoter and enhancer elements of the *ER* gene, and depletion of the enzyme in BCa cells elevates H3K9 methylation and attenuates GATA-3 association at these sites, thus reducing receptor expression. Our data highlight a novel role for KDM4B in the ER signalling cascade and demonstrates a cross-regulatory network involving KDM4B, ER and GATA-3 that is required for *ER* gene expression. Lastly, we find that KDM4B also interacts with and regulates FOXA1 expression in BCa cells, indicating an additional means of regulating the activity of the ER. In all, our findings support the hypothesis that by regulating *ER* gene transcription, pioneer factor activity and facilitating estrogen-dependent gene expression, KDM4B is a master regulator of receptor signalling and confirms this demethylase as a potentially important target in BCa therapy.

## MATERIALS AND METHODS

### Plasmids and antibodies

The following plasmids have been previously described: pCMV-ER, MUC1-responsive reporter (kind gift from Prof. Vincent Giguere, McGill University) (18), pCMV-GATA-3 (kind gift from Dr Jason Carroll, Cambridge Research Institute, UK) and pCMV-HA-KDM4B and pCMV-HA-KDM4B_H189G/E191Q_ (kind gift from Dr Peter Staller, BRIC, Denmark) (19). Antibodies for ER (D-20) and GATA-3 (HG3-31) were purchased from Santa Cruz Biotechnology, KDM4B (A301-478A) from Bethyl Laboratories and FOXA1 (Ab 23738) from Abcam. Antibodies to histone methylation/acetylation marks have been previously described (20). To generate pGL3-Enh1 and pGL3-Enh2 reporters, we amplified ∼500 bp *ER* gene Enh1 and Enh2 fragments from MCF-7 DNA with primers containing *Xho* 1 and *Hind* III restriction sites (see Supplementary Table S1), digested with indicated enzymes and ligated directionally into pGL3-basic (Promega).

### Cell culture and DNA transfection

Cell culture and DNA transfection were performed as described previously (21). MCF-7, T47D and BT-474 cells were maintained in RPMI-1640 media containing 10% foetal calf serum (FCS) (Sigma). The estrogen-independent MCF-7 (EI-MCF-7) cell line was generated by continuous culturing of MCF-7 cells in phenol red–free RPMI supplemented with 10% steroid-depleted FCS (Hyclone) for 6 months before characterization. For reporter assays, 1 × 10^4^ MCF-7 cells were routinely plated per well in 24-well microtitre plates (Corning). After 24 h, the cells were transfected for 48 h using LT-1 reagent (Mirus Bio) according to the manufacturer’s recommendations and were subsequently harvested and assayed for luciferase and β-galactosidase activity as described previously (22). For estrogenic stimulation or 4-hydroxytamoxifen (Tam) treatment experiments, cells were grown in phenol red–free RPMI-1640 media supplemented with 10% serum-stripped FCS (Hyclone) before transfection as above. Either 8 or 24 h before harvesting for mRNA extraction [as described in (20)] or luciferase assays, respectively, cells were treated with or without 10 nM β-estradiol (E_2_) (Sigma) or 1 µM 4-hydroxytamoxifen (Sigma). Unless indicated, quantitative polymerase chain reaction (PCR) analysis data of ER and ER-target gene mRNA expression [performed as described in (20); primers for each are shown in Supplementary Table S1] and luciferase assay data represents an average of three repeats ± standard error (*denotes *P* < 0.05). For western blot analysis, parallel experiments to those described above were harvested in sodium dodecyl sulphate (SDS)-sample buffer and subject to polyacrylamide gel electrophoresis (SDS-PAGE) before immunoblotting with specific antibodies [as described in (23)]. Cycloheximide time-course experiments were conducted as described in (20). Proliferation assays using WST-1 (Roche) were performed as described in (20), and BrdU incorporation assays were performed according to the manufacturer’s recommendations (Roche). BCa cells were pretreated for 3 h with a final concentration of 1 µM fulvestrant (Sigma) before E_2_ stimulation for an additional 45 min before subsequent gene expression or chromatin immunoprecipitation (ChIP) analysis.

### siRNA transfection

All siRNAs used in the study were purchased from Sigma, and sequences are shown in Supplementary Table S2. To rule out off-target effects of KDM4B and GATA-3 knock-down, MCF-7, T47D and BT-474 BCa cell lines were transfected with four KDM4B siRNAs [labelled siKDM4B (A)–(D)] either individually or as a pool consisting of siKDM4B (A)/(B)/(C), or two GATA-3 [labelled siGATA-3 (1)/(2)] oligonucleotides using Lipofectamine RNAiMax (Invitrogen) according to manufacturer’s recommendations at a final concentration of 25 nM and as described (20). Unless otherwise stated, KDM4B knock-down experiments were primarily performed with siKDM4B (C) oligonucleotides. The initial siRNA library screen was conducted by reverse transfecting MCF-7 cells grown in phenol red–free steroid-depleted media for 72 h in the presence and absence of 10 nM E_2_ with a pool of three individual siRNAs per 50 HMT and 27 HDM enzymes studied. mRNA was extracted using Trizol and cDNA generated and analysed by quantitative PCR, as described (20). Additional transcriptional validation experiments were performed using individual or pooled siKDM4B (A)/(B)/(C) compared with cells depleted of ER or pS2 (see Supplementary Table S2 for sequences).

For RNAi rescue experiments, five silent mutations were introduced into the KDM4B siRNA (C) target sequence within pCMV-HA-KDM4B by two rounds of site-directed mutagenesis using the following primer combinations: KDM4B-CS1 F: 5′-TGC GAC GCC TTC CTG CGA CAC AAA ATG ACC CTC ATC TGC-3′, KDM4B-CS1 R: 5′- GCA GAT GAG GGT CAT TTT GTG TCG CAG GAA GGC GTC GCA-3′ and KDM4B-CS2 F: 5′-CTG CGA CAC AAA ATG ACA CTG ATC TCG CCC ATC-3′, KDM4B-CS2 R: 5′-GAT GGG CGA GAT CAG TGT CAT TTT GTG TCG CAG-3′, to permit expression of ectopic enzyme in cells depleted of endogenous KDM4B. The resultant ‘codon-switch (CS)’ pCMV-HA-CS-KDM4B vector or control plasmid was co-transfected with either scrambled (Scr) or KDM4B (C) siRNAs for 48 h before respective western blot and quantitative PCR analyses of ER protein and mRNA levels.

### Immunoprecipitation, ChIP and chromatin fractionation

To investigate endogenous KDM4B-ER and KDM4B-GATA-3 interactions in MCF-7 and T47D cells, immunoprecipitations (IPs) were performed using KDM4B, ER and GATA-3 antibodies using a previously described protocol (21). To assess ectopically expressed KDM4B-ER and KDM4B-FOXA1 interactions, pCMV-HA-KDM4B was transiently transfected into MCF-7 cells, and after 48 h, resultant ER or FOXA1 immunoprecipitates were subject to western blotting using a KDM4B antibody.

ChIP was performed essentially as described (24). Briefly, 2 × 10^6^ MCF-7 or T47D cells were cultured in steroid-depleted media for a total of 72 h including a 10 nM E_2_ treatment for the last 45 and 180 min of incubation. Cells were fixed in 1% formaldehyde for 10 min and glycine treated for 5 min before cell scraping and centrifugation. Resultant pellets were washed in phosphate buffered saline (PBS) and resuspended in LB1 solution (50 mM HEPES-KOH, pH 7.5; 140 mM NaCl; 1 mM EDTA; 10% glycerol; 0.5% NP-40; 0.25% Triton-X-100) for 10 min with gentle agitation at 4°C. Samples were centrifuged at 1500*g* for 5 min before lysis in 10 ml LB2 buffer (10 mM Tris–HCl, pH 8; 200 mM NaCl; 1 mM EDTA; 0.5 mM EGTA) at 4°C for 10 min with gentle agitation. Lysates were centrifuged as before and resultant pellets resuspended in 0.5 ml LB3 buffer (10 mM Tris–HCl, pH 8; 100 mM NaCl; 1 mM EDTA; 0.5 mM EGTA; 0.1% Na-Deoxycholate; 0.5% *N*-lauroylsarcosine). Samples were subject to a preoptimized sonication programme using a Bioruptor^TM^ (Diagenode) incorporating 20 cycles of 30 s on/30 s off at the ‘Hi’ setting and then centrifuged for 10 min at 13 000*g*. DNA concentration for each supernatant was measured using a Nanodrop spectrometer, and 100 μg chromatin was transferred into a fresh tube and diluted 5-fold in LB3 buffer containing 1% Triton-X-100. Ten percent of each solution was taken as input and frozen until the next day. Fifty microlitre per sample of Dynabeads conjugated to Protein A (Invitrogen) was transferred into fresh tubes and blocked with three washes of PBS containing 0.5% bovine serum albumin (PBS-BSA) before incubation with 2 μg antibodies overnight at 4°C. Dynabeads were then washed twice with PBS-BSA to remove any unbound antibody, and the 50 μg chromatin samples prepared the previous day were added to the appropriate Dynabead samples and mixed overnight at 4°C. Dynabeads were subsequently washed six times in RIPA buffer (50 mM HEPES-KOH, pH 7.5; 500 mM LiCl; 1 mM EDTA; 1% NP-40; 0.7% Na-Deoxycholate) and once in Tris-buffered saline (20 mM Tris–HCl, pH 7.6; 150 mM NaCl) before incubation for 8 h in 200 μl elution buffer (50 mM Tris–HCl, pH 8; 10 mM EDTA) at 65°C to elute protein–DNA complexes and to reverse formaldehyde-induced cross-links. In addition, input samples taken the previous day were defrosted and subject to elution/cross-link reversal. Samples were diluted 1-fold in TE buffer and subject to proteolytic digestion using 4 μl 20 mg/ml proteinase K (Invitrogen) for 1 h at 55°C. DNA was purified using a GeneElute^TM^ genomic DNA miniprep kit (Sigma) and subject to quantitative PCR using primers specific to *cis*-regulatory elements of the *pS2* (*TFF1*), *GREB1* and *ER* genes (Supplementary Table S1). Data were presented as % Input using the following formula: % Input = 100 × 2^(CT Adjusted Input sample − CT immunoprecipitated sample) (CT refers to cycle threshold). At least two individual repeats of the entire experiment were conducted and data combined to give an average % Input (* denotes *P* < 0.05).

For Re-ChIP experiments, protein–DNA complexes from the first round of ChIP using a GATA-3 antibody were eluted in 10 mM DTT and diluted 10 × in LB3 buffer and subject to ChIP with either anti-KDM4B or isotype control antibodies.

To assess the role of KDM4B at *cis*-regulatory elements of the *ER*, *pS2*, *GREB1* and *FOXA1* genes, MCF-7, EI-MCF-7 and T47D cells were transiently transfected with either Scr or KDM4B siRNAs in steroid-depleted phenol red–free media and treated with 10 nM E_2_ for 45 min before formaldehyde cross-linking. Antibodies to KDM4B, GATA-3 and histone methylation and acetylation marks were used in ChIP as described above.

Chromatin fractionation was performed as described (25) using 2 × 10^6^ MCF-7 and T47D cells. Resultant fraction 3, representing the chromatin-bound fraction of protein, was subject to SDS-PAGE and immunoblotted with antibodies as indicated in the figure legends. Acid extraction of histone proteins was conducted using the protocol outlined in (20).

## RESULTS

### Confirming the involvement of KDM4B in the ER signalling cascade

**Figure 1.**
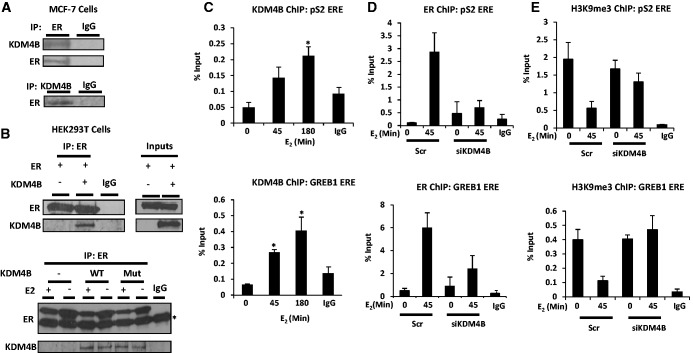
KDM4B interacts with the ER and is recruited to hormone-responsive genes. (**A**) MCF-7 cells grown in serum-containing media were subject to IP using anti-ER, anti-KDM4B or control antibodies before western blot analysis using reciprocal antibodies. (**B**) HEK293T cells grown in either serum-containing or steroid-depleted media supplemented with 10 nM E_2_ were transiently transfected with mammalian expression vectors encoding ER and wild-type/demethylase-dead KDM4B (Mut) or empty vector control for 48 h before IP as in (A) (* denotes a non-specific IgG band). (**C**) MCF-7 cells were grown in steroid-depleted media for 48 h and treated with 10 nM E_2_ for 45 and 180 min before ChIP analysis using an anti-KDM4B antibody and analysis of recruitment to *pS2* and *GREB1* estrogen-responsive elements (EREs) by quantitative PCR. (**D**) MCF-7 cells were transiently transfected with either Scr control or KDM4B siRNA (siKDM4B) in steroid-depleted media for 72 h with or without 45 min 10 nM E_2_ stimulation before ChIP with an anti-ER antibody or an anti-H3K9me3 antibody (**E**), and subsequent analysis at the *pS2* and *GREB1* EREs. ChIP data are an average of three independent experiments ± standard error (* denotes *P* < 0.05).

To identify HMT and HDM enzymes regulating the ER in BCa cells, an unbiased siRNA library screen was performed in which each of the human HMT and HDM enzymes were individually depleted in MCF-7 cells, and transcriptional activity of the receptor was assessed by measuring *pS2* (*TFF1*) gene expression (data not shown). Consistent with two reports describing that the H3K9me3/me2 demethylase KDM4B as a regulator of ER activity (14,15), our experiments using several siRNAs [siKDM4B (A)/(B)/(C)], either individually or pooled, confirmed that KDM4B depletion down-regulated expression of ER-dependent *pS2*, *progesterone receptor* (*PgR*) (Supplementary Figures S1A/B) and *GREB1* genes (Supplementary Figure S10A) in response to 10 nM E_2_. This effect was specific to KDM4B, as knock-down of other KDM4 family members KDM4A, KDM4C and KDM4D did not affect ER-mediated transcription (data not shown). We next sought to determine if KDM4B and ER interacted by probing endogenous ER and KDM4B immunoprecipitates from MCF-7 cells with the reciprocal antibodies. As previously reported (14), we found an interaction between the two proteins (Figure 1A) and confirmed this by IP in HEK293T cells ectopically expressing wild-type KDM4B or demethylase-dead KDM4B_H189G/E191Q_ and the ER (Figure 1B). Using ChIP in MCF-7 cells, we show that KDM4B is recruited to ER-responsive elements within the proximal promoter regions of *pS2* and *GREB1* genes on E_2_ stimulation (Figure 1C) that coincides with ER recruitment and reduced enrichment of H3K9me3 marks (Supplementary Figure S2). Depletion of the enzyme using siKDM4B (C) attenuates ligand-dependent recruitment of the ER (Figure 1D) and up- and down-regulates respective H3K9me3 (Figure 1E) and H3K9 acetylation marks (Supplementary Figure S3) at these loci, consistent with reduced gene expression. Global histone modification marks were also analysed in cells depleted of KDM4B and the findings are in-keeping with previous reports (16), describing effects to H3K9 methylation and acetylation marks (Supplementary Figure S4). These findings are consistent with previous reports and confirm an involvement of KDM4B in the ER signalling cascade.

### KDM4B depletion reduces ER gene expression

**Figure 2.**
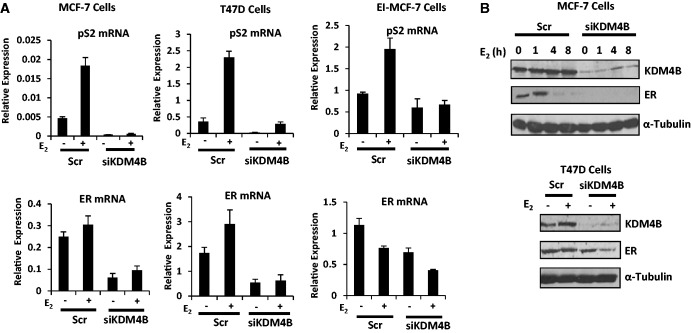
KDM4B regulates *ER* gene expression. (**A**) MCF-7, T47D and EI-MCF-7 cell lines were subject to transient transfection with either Scr control or KDM4B (siKDM4B) siRNAs in steroid-depleted media for 40 h before 8-h 10 nM E_2_ stimulation and subsequent RNA extraction. Resultant cDNA was analysed for pS2 and ER expression. Data are the average of three independent experiments performed in triplicate ± standard error. (**B**) Parallel experiments to (A), but cells were harvested in SDS-sample buffer before western blot analysis using antibodies to KDM4B, ER and α-Tubulin.

Unexpectedly, during the course of our experiments, we observed that KDM4B depletion by siKDM4B (C) markedly reduced ER mRNA and protein levels irrespective of estrogen status in several KDM4B expressing BCa cell lines, including T47D and both MCF-7 and an estrogen-dependent variant of this cell line, EI-MCF-7 (see ‘Materials and Methods’ section) (Figure 2, Supplementary Figure S5–7), a finding that had not been reported in the previous studies (14,15). We predict that this reduction in ER protein is exclusively a consequence of down-regulated *ER* gene expression and not enhanced protein turnover, as cycloheximide time-course experiments in MCF-7 cells ectopically expressing wild-type KDM4B or KDM4B_H189G/E191Q_ failed to show an impact on ER stability (Supplementary Figure S8, middle and right panel). Interestingly, we find that endogenous KDM4B has a rapid half-life (<1 h), suggesting tight cellular control over this enzyme (Supplementary Figure S8, left panel).

To rule out the possibility that these findings were off-target effects of KDM4B depletion by one oligonucleotide, we firstly used two additional KDM4B-targeting siRNAs [siKDM4B (A) and (B)] and showed that in addition to attenuated pS2 expression, ER mRNA and protein levels were also down-regulated in MCF-7 (Supplementary Figure S9A), BT-474 (Supplementary Figure S9B) and T47D (data not shown) BCa cell lines. We next incorporated an additional siRNA as described in the Shi *et al.*, study (14) [denoted siKDM4B (D)] into ER expression profiling experiments and showed that receptor levels, as well as pS2 and GREB1 mRNA, were again depleted in MCF-7 cells grown in steroid-depleted media supplemented with and without 10 nM E_2_ (Supplementary Figure S10A). This finding was replicated in MCF-7 and T47D cells grown in serum-containing media (Supplementary Figure S10B) confirming that reduced ER expression is a genuine phenotype of KDM4B-depleted BCa cells. Moreover, growth assays conducted in both MCF-7 and T47D cells demonstrated reduced cell proliferation and a subtle, but significant, increase in apoptosis in response to KDM4B knock-down, suggesting reduced ER levels is likely to be causative for the aberrant phenotype of the BCa cells (Supplementary Figure S11A and S11B). Importantly, these data suggest that reduced ER recruitment to target genes, dys-regulated histone methylation/acetylation marks and reduced receptor-mediated transcription evident on KDM4B depletion [as shown in (14,15), Figure 1 and Supplementary Figures S1 and S3] may be largely ascribed to reduced ER protein levels and challenges the concept of KDM4B as a *bona fide* ER co-activator.

### KDM4B interacts with and co-activates GATA-3

**Figure 3.**
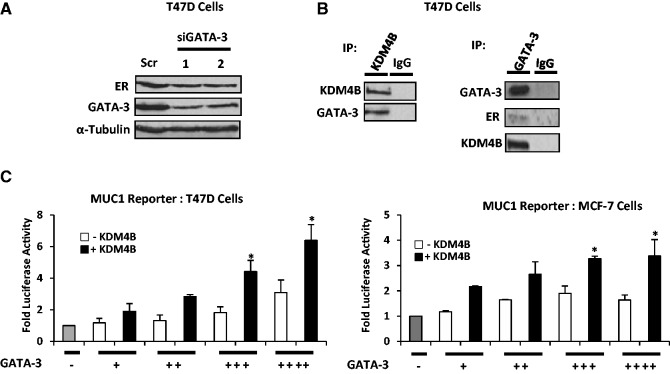
KDM4B is a co-regulator of GATA-3. (**A**) T47D cells grown in serum-containing media were depleted of GATA-3 using two individual siRNAs [labelled siGATA-3 (1) and (2)] for 48 h before western blot analysis using antibodies to ER, GATA-3 and α-Tubulin. Scr control siRNA was used as a control. (**B**) T47D cells grown in serum-containing media were subject to IP using antibodies to either KDM4B, GATA-3 or isotype control, and interactions using reciprocal antibodies were assessed by western blot analysis. (**C**) T47D or MCF-7 cells grown as above were transiently co-transfected with a GATA-3-responsive MUC1 luciferase reporter and KDM4B and increasing amounts of GATA-3 mammalian expression vectors. After 48 h, cells were harvested for luciferase and β-galactosidase activities, and data are representative of N = 3 experiments ± standard error (* denotes *P* < 0.05).

**Figure 4.**
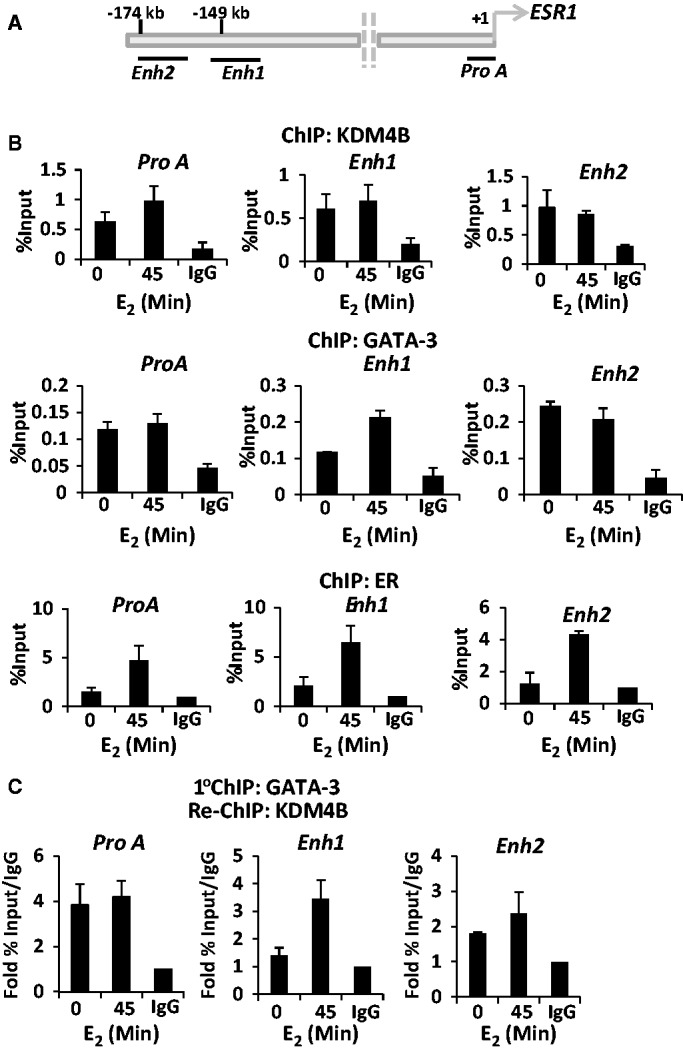
KDM4B is recruited to *cis*-regulatory elements of the *ER* gene. (**A**) Diagrammatic representation of the position of the upstream regulatory elements of the *ER* gene including Pro A, enhancer 1 (Enh1) and enhancer 2 (Enh2) in respect to the transcriptional start site (+1). (**B**) T47D cells were grown in steroid-depleted media for 48 h and treated with 10 nM E_2_ for 45 min before ChIP analysis using anti-KDM4B, -GATA-3 and –ER antibodies or isotype control. Recruitment to Pro A, Enh1 and Enh2 was assessed by quantitative PCR. Data are an average of three independent experiments ± standard error. (**C**) Cells were grown and treated as in (B) and then subject to a first-round ChIP using an anti-GATA-3 antibody. Eluted complexes were then introduced into a second round of ChIP using either anti-KDM4B or isotype control antibodies, and resultant DNA was analysed by quantitative PCR using primers to Pro A, Enh1 and Enh2 regions. Data are the average of three independent experiments ± standard error.

To address how KDM4B controls *ER* gene expression, we hypothesized that the enzyme was working through a known regulator of *ER* transcription to facilitate up-regulation of receptor levels in BCa cells. The transcription factor GATA-3 has been well documented to drive expression of the ER, and as such GATA-3 expression is highly associated with ER-positive BCa (26,27). Importantly, GATA-3 has been reported to directly bind the upstream promoter A (Pro A) and enhancer element 1 (Enh1) and -2 (Enh2) of the *ER* gene (Figure 4A), and depletion of the transcription factor in T47D and MCF-7 cells reduces ER levels, indicating a direct role for GATA-3 in regulating expression of the receptor [(17), Figure 3A, Supplementary Figure S12]. The comparable effects of GATA-3 and KDM4B knock-down on ER expression suggested that the two proteins may function as part of the same transcriptional regulatory complex. To test this, we performed IP of KDM4B in T47D cells and showed that GATA-3 was specifically associated with the demethylase, but not from an immunoprecipitate using an isotype-control antibody (Figure 3B, left panel). To confirm the interaction, the reciprocal experiment was performed and we detected KDM4B in GATA-3 immunocomplexes from T47D cells, but again not in the control experimental arm (Figure 3B, right panel), indicating that the two proteins are associated in BCa cells.

To address whether KDM4B is a co-regulator of GATA-3, we performed reporter experiments in both T47D and MCF-7 cells, incorporating a GATA-3-responsive MUC1 promoter element upstream from the *luciferase* gene (18). As expected, increasing amounts of GATA-3 increased activity of the reporter by up to 3-fold (Figure 3C, white bars), and this was markedly increased by overexpression of KDM4B (Figure 3C, black bars), indicating that KDM4B co-activates GATA-3 in this system. Further to this, we cloned 500 bp fragments encompassing Enh1 and Enh2 regions of the *ER* gene into a luciferase reporter plasmid and measured activity in the presence and absence of GATA-3 and KDM4B. In MCF-7 cells, GATA-3 selectively up-regulated the Enh2 reporter that was further elevated by ectopic expression of KDM4B, confirming the role of the demethylase in co-activating GATA-3 (Supplementary Figure S13A). This was further confirmed in ER negative HEK293T cells (Supplementary Figure 13B).

### KDM4B is present at *ER* gene *cis*-regulatory elements in complex with GATA-3

To decipher the mechanism of KDM4B-mediated regulation of GATA-3 activity in a more physiologically relevant model, we firstly examined KDM4B recruitment upstream of the *ER* gene in the presence and absence of 10 nM E_2_ by ChIP in T47D cells. Previous reports have shown that both GATA-3 and the ER are associated with Enh1, Enh2 and pro A elements of the upstream regulatory regions of the *ER* gene [(17), Figure 4A], and thus we focused our experiments on these loci. As shown in Figure 4B (upper panel), KDM4B is associated with all three *cis*-regulatory elements in the absence of estrogen, demonstrating ∼5-fold enrichment over isotype antibody control, and remains present after-45 min hormone stimulation. As a control, KDM4B was not associated with a distal site lacking GATA-3 or ER-binding sites (data not shown). Importantly, we show that GATA-3 reciprocates the chromatin-binding kinetics of KDM4B and is present constitutively at the enhancer and promoter regions of the *ER* gene (Figure 4B, middle panel), suggesting the formation of a complex between the demethylase and GATA-3 at these loci.

To examine this further, we performed re-ChIP experiments in T47D cells in which GATA-3 immunocomplexes were re-immunoprecipitated with an anti-KDM4B antibody, and resultant DNA analysed by quantitative PCR using primer sets to Pro A, Enh1 and Enh2 sites. As shown in Figure 4C, GATA-3 and KDM4B are associated in the same complex at these *cis*-regulatory elements, confirming the notion that the two proteins function together to regulate ER expression. Interestingly, the ER is also recruited to the three *cis*-regulatory sites of the *ER* gene in response to estrogen (Figure 4B, lower panel) and has been shown to be co-recruited with GATA-3 to these regions (17), suggesting that the enhancement of the GATA-3-KDM4B interaction at Enh1 in the presence of hormone (Figure 4C) may simply be a consequence of elevated GATA-3 recruitment (Figure 4B, middle panel).

Given the presence of the ER at its own promoter and enhancer regions, one important consideration is whether reduced expression of the *ER* gene on depletion of KDM4B is simply a consequence of attenuated KDM4B-mediated co-activation of the receptor itself that may down-regulate ER levels independently of GATA-3 function. To address this issue, we performed *ER* gene expression analysis in both MCF-7 and T47D cells transiently transfected with KDM4B oligonucleotides [siKDM4B (C) and (D)] in the presence of estrogen supplemented with and without 1 µM 4-hydroxy-tamoxifen (Tam). We speculated that if chromatin-bound ER was an important target of KDM4B-mediated co-activation activity on the *ER* gene, there would be reduced receptor expression in both the Tam-treated and KDM4B-depleted arms of the experiment. Predictably, Tam treatment reduced expression of *pS2* in both MCF-7 and T47D cells (Supplementary Figure S14A and S14B, left panels). Importantly, Tam supplementation alone failed to appreciably affect ER mRNA levels compared with control. In contrast, Tam-treated cells depleted of KDM4B exhibited a marked reduction in ER mRNA levels equivalent to the ER knock-down control, indicating that KDM4B is likely functioning through GATA-3, and not via the receptor, to regulate *ER* gene transcription (Supplementary Figure S14A and S14B, right panels). To further support this hypothesis, we reasoned that depletion of ER in BCa cells by treatment with the SERD fulvestrant would not affect recruitment of KDM4B to *ER* gene *cis*-regulatory elements. To firstly demonstrate the efficacy of fulvestrant in MCF-7 cells, we showed that 3-h fulvestrant pretreatment reduced ER protein levels and attenuated estrogen-induced recruitment of the ER to target genes *pS2* and *GREB1* (Supplementary Figures S15A and S15B). Importantly, fulvestrant had no significant effect on KDM4B recruitment to the promoter and enhancer elements of the *ER* gene (Supplementary Figure S15C). The marginal reduction in KDM4B promoter association at Enh1 on fulvestrant treatment is likely a result of reduced KDM4B levels due to the demethylase being a direct ER target gene (Supplementary Figure S15B, middle panel).

### KDM4B depletion elevates H3K9me3 marks and reduces GATA-3 binding to the *ER* gene

**Figure 5.**
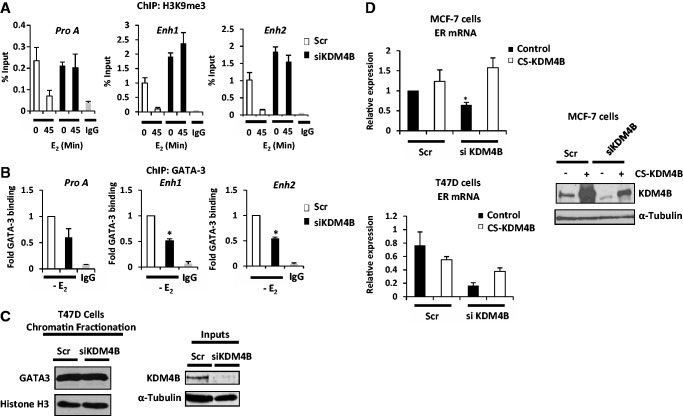
KDM4B regulates H3K9 methylation and GATA-3 binding at the *ER* gene promoter and enhancers. T47D cells were transfected with Scr control or KDM4B (siKDM4B) siRNAs in steroid-depleted media for 48 h and treated with 10 nM E_2_ for 45 min before ChIP analysis using anti-H3K9me3 (**A**) or -GATA-3 antibodies (**B**). Resultant DNA was analysed by quantitative PCR using primers to Pro A, Enh1 and Enh2 regions, and data shown are the average of three independent experiments ± standard error (* denotes *P* < 0.05). (**C**) T47D cells grown in serum-containing media were transfected with Scr control or siKDM4B siRNAs for 48 h before chromatin fractionation. Resultant lysates were subject to western blot analysis using anti-GATA-3 and -histone H3 antibodies. Input samples were run alongside to demonstrate depleted KDM4B levels in response to siRNA transfection. (**D**) MCF-7 and T47D cells were co-transfected with siRNAs (Scr or siKDM4B) and mammalian expression vectors (CS-KDM4B or empty vector control) for 48 h in serum-containing media before RNA extraction and ER mRNA expression profiling by quantitative PCR. Data are the average of three experiments performed ± standard error (*P < 0.05). Parallel experiments were harvested in SDS-samples buffer and subject to western blot analysis using anti-KDM4B and α-Tubulin antibodies.

The binding of KDM4B to both enhancer and promoter elements suggest it may have a role in modifying the chromatin landscape at these sites to facilitate GATA-3-mediated transcription of the *ER* gene. To address this, we firstly performed ChIP experiments using an anti-histone H3K9me3 antibody in T47D cells transfected with and without siKDM4B (C) oligonucleotides and assessed flux to this repressive mark in response to 10 nM E_2_ at Pro A, Enh1 and Enh2 loci. As shown in Figure 5A (white bars), a marked decrease in H3K9me3 was observed in response to 45-min estrogen treatment consistent with transcriptional activation. In cells depleted of KDM4B, however, no decrease in H3K9me3 marks was observed in response to hormone stimulation consistent with reduced *ER* expression. Moreover, consistent with effects of KDM4B knock-down on basal ER mRNA expression (Figure 2 and Supplementary Figure S6), enrichment of H3K9me3 was increased at both Enh1 and Enh2 regions in the absence of hormone on KDM4B knock-down, confirming a role for KDM4B as a regulator of ER expression independently of the receptor (Figure 5A, black bars). We also investigated the effect of KDM4B depletion on H3K9 methylation of the *ER* gene cis-regulatory elements in the EI-MCF-7 cell line. In contrast to the consistent estrogen-dependent reduction in H3K9me3 marks at Pro A, Enh1 and Enh2 in T47D cells, only Enh1 and Enh2 loci demonstrated a reduction in repressive methylation marks on estrogen stimulation that was attenuated on KDM4B knock-down (Supplementary Figure S16). Intriguingly, contrary to data presented in Figure 2A, demonstrating a dependency on KDM4B for *ER* expression in EI-MCF-7 cells, basal H3K9me3 was markedly reduced on KDM4B depletion at both Enh1 and Enh2 elements (Supplementary Figure S16), suggesting there may be a partial uncoupling of the repressive methylation mark and its effect on transcriptional output of the *ER* gene in long-term steroid-depleted conditions.

We next assessed the effect of KDM4B knock-down on GATA-3 association at the promoter and enhancer elements of the *ER* gene by ChIP in T47D cells. Given that our experiments demonstrate constitutive GATA-3 binding at Pro A, Enh1 and Enh2 loci (Figure 4B, middle panel), we conducted ChIP experiments in the absence of estrogen. At each of the *cis*-regulatory elements of the ER gene, we observed that GATA-3 association is reduced by ∼50% in cells depleted of KDM4B, indicating an important role for KDM4B in facilitating GATA-3 binding (Figure 5B). Reduced GATA-3 chromatin association was not a consequence of reduced cellular GATA-3 protein levels as shown by western blot analysis of lysates from MCF-7 and T47D cells subject to KDM4B knock-down (Supplementary Figure S17). Moreover, chromatin fractionation experiments [as described in (25)] demonstrated that global GATA-3 chromatin association is not attenuated on KDM4B depletion (Figure 5C), implying that loss of GATA-3 from the ER promoter and enhancer loci is a consequence of localized chromatin remodelling and not a genome-wide phenomenon.

To further provide evidence for KDM4B regulating *ER* gene transcription, we performed RNAi-rescue experiments in which MCF-7 and T47D were transiently transfected with siKDM4B (C) together with either a KDM4B cDNA resistant to siRNA-mediated depletion, termed CS-KDM4B (see ‘Materials and Methods’ section) or empty vector control. As expected, KDM4B knock-down reduced ER expression in both BCa cell lines (Figure 5D, black bars), albeit less in MCF-7 cells compared with data presented in Figure 2A that may reflect differences in the transfection procedure. In contrast, overexpression of CS-KDM4B had minor effect on ER mRNA levels in the presence of Scr siRNA control (Figure 5D, compare lanes 1 and 2). However, ectopic expression of CS-KDM4B in cells depleted of the demethylase was able to fully rescue receptor expression in MCF-7 cells and by >50% in T47D cells, indicating a genuine role for KDM4B in controlling transcription of the *ER* gene (Figure 5D, compare lanes 2 and 4).

### KDM4B interacts with and regulates FOXA1 expression

**Figure 6.**
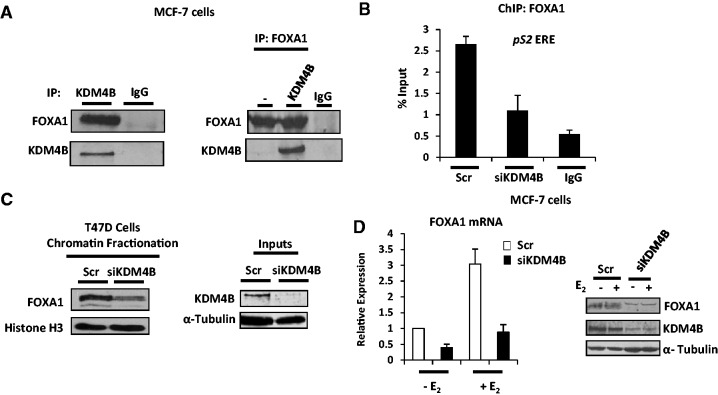
KDM4B interacts with and regulates expression of FOXA1. (**A**). MCF-7 cells grown in serum-containing media were subject to IP using either anti-KDM4B or isotype control antibodies and resultant samples analysed by western blot analysis using FOXA1 and KDM4B immunoglobulins (left panel). MCF-7 cells were also transiently transfected with or without KDM4B mammalian expression vectors for 48 h before IP using an anti-FOXA1 antibody (right panel). Samples were analysed as before. (**B**) MCF-7 cells were transiently transfected with either Scr or KDM4B (siKDM4B) siRNAs in serum-containing media for 48 h before ChIP analysis using anti-FOXA1 or isotype control antibodies. Data are the mean of three independent experiments analysing FOXA1 recruitment to the pS2 gene promoter ERE. (**C**) T47D cells were transiently transfected with either Scr or KDM4B (siKDM4B) siRNAs in serum-containing media for 48 h before chromatin fractionation and western blot analysis using anti-FOXA1 and α-Tubulin antibodies. (**D**) MCF-7 cells were transiently transfected with either Scr control or KDM4B (siKDM4B) siRNAs in steroid-depleted media for 40 h before 10 nM estrogen treatment for 8 h. RNA was extracted from samples, and resultant cDNA was used in quantitative PCR to assess FOXA1 expression levels (left panel). Parallel experiments were performed as described above and samples lysed in SDS-sample buffer before western blot analysis using FOXA1, KDM4B and α-Tubulin antibodies (right panel).

The importance of the pioneer factor FOXA1 in regulating ER-mediated transcription is underscored by the high proportion of receptor–target gene enhancer elements containing FOXA1-binding forkhead motifs and the requirement for deposition of FOXA1 at these regions to facilitate active ER recruitment (25,28,29). Our current understanding of selective FOXA1–chromatin association is limited, but evidence suggests that regions depleted of H3K9me2 and enriched for H3K4me2 demarcate binding loci for this pioneer factor (25). Given that KDM4B is a regulator of ER activity and demethylates H3K9me3, we hypothesized that KDM4B may regulate the discriminate deposition of FOXA1 and hence participate in the global control of receptor signalling. To this end, we firstly examined if FOXA1 and KDM4B interacted by IP using an anti-KDM4B antibody in MCF-7 cells. As shown in Figure 6A (left panel), FOXA1 is present in KDM4B immunoprecipitates, but not in the isotype antibody control. Performing the reciprocal experiment, we demonstrated successful KDM4B co-IP from a FOXA1 immunocomplex in MCF-7 cells ectopically expressing KDM4B (Figure 6A, right panel), indicating that the proteins are in complex and could function together.

To address the role of KDM4B in regulating FOXA1 chromatin association, we performed ChIP using an anti-FOXA1 antibody in MCF-7 cells depleted of KDM4B and assessed binding of FOXA1 to the *pS2* gene enhancer. In cells transfected with Scr siRNA, we detected 5-fold enrichment of FOXA1 binding over isotype antibody control that was markedly reduced on knock-down of the demethylase (Figure 6B). Using chromatin fractionation, we further showed that depletion of KDM4B robustly reduced global binding of FOXA1 to chromatin (Figure 6C). Importantly, however, assessment of FOXA1 levels in MCF-7 cells depleted of KDM4B demonstrated reduced expression of the pioneer factor in the presence and absence of estrogen that was a direct consequence of reduced mRNA expression (Figure 6D, left panel) and not an effect on protein stability as measured by cycloheximide time-course experiments (Supplementary Figure S18).

To provide additional evidence that KDM4B was a regulator of basal and estrogen-induced *FOXA1* expression, we showed that the demethylase was associated at three upstream elements of the *FOXA1* gene (Supplementary Figure S19A) in the absence of estrogen and was reduced on hormonal stimulation (Supplementary Figure S19B), suggesting an important role for KDM4B in priming FOXA1 transcription. Importantly, we found that repressive H3K9me3 marks at these loci were reduced in response to estrogen, but not in MCF-7 cells depleted of KDM4B, implicating the enzyme in controlling expression of *FOXA1* in both basal and estrogenic conditions (Supplementary Figure S19C).

Given the interplay between GATA-3, ER, FOA1 and KDM4B, we lastly wanted to establish if FOXA1 was recruited to *ER* gene Pro A, Enh1 and Enh2 *cis*-regulatory elements, as this could provide an additional level of KDM4B-mediated regulation of the entire ER signalling network. As shown in Supplementary Figure S20, we find that FOXA1 is recruited to the *ER* promoter and enhancer elements in response to 45-min estrogen stimulation that is markedly reduced by 180-min treatment, suggesting FOXA1 has a potential role in regulating expression of the receptor.

Together, the data suggest that KDM4B regulates ER activity at multiple levels, including controlling ER expression and activity, and expression of FOXA1, a critical component of the receptor signalling cascade.

## DISCUSSION

The expanding repertoire of co-regulator and pioneer factor proteins required for ER activity identified over the past decade has provided an indication of the mechanistic complexity required for acute transcriptional control of the receptor. Importantly, deregulated activity of several co-regulator proteins has been postulated to facilitate the transition from hormone-naïve to endocrine-therapy-resistant BCa (3), suggesting future therapies for breast malignancy may target additional components of the ER signalling cascade.

One such ER co-regulator, the HDM enzyme KDM4B, has been shown in two previous studies to be a requisite for transcriptional activation of several receptor-target genes by demethylating repressive histone H3 lysine 9 tri-/di-methyl marks at gene promoter and enhancer elements (14,15). In the present study, we have confirmed that KDM4B is a regulator of the ER signalling cascade, but has challenged the concept that it is a direct ER co-activator working exclusively to enhance transcription of receptor–target genes. Instead, our data demonstrates that KDM4B is a key regulator of *ER* gene transcription and *FOXA1* expression that together control the estrogen-dependent phenotype of a high proportion of BCa cell lines and primary tumours. Hence, KDM4B-depletion experiments demonstrating reduced *pS2* and *GREB1* expression, as well as attenuated proliferation in BCa cell lines [(14,15), Supplementary Figures S1, S6, S9, S11 and Figure 2], is likely a consequence of down-regulated ER and FOXA1 levels that ultimately abrogates the global receptor signalling network. Critically, one important issue raised by our experiments is the discrepancy between the reporting of ER protein levels on KDM4B depletion by ourselves and the two previously published studies (14,15). Using a total of four siRNAs, including one used in the Shi *et al.* study (14), we have demonstrated in multiple BCa cell lines grown in both steroid-containing and -depleted conditions that reduced ER mRNA expression is a consistent phenotype of KDM4B knock-down. Importantly, the Shi *et al.* study demonstrates KDM4B association to the upstream regulatory elements of the *ER* gene (14), consistent with our findings in Figure 4, suggesting it is a genuine regulator of receptor expression. Although difficult to conclude, the differences may have arisen through subtle variation in experimental parameters or detection that may occlude the identification of changes to ER levels in the aforementioned study (14).

GATA-3 is a well-characterized regulator of ER expression (17), but how it functions to directly control transcription is not well defined. Given the complex nature of ER gene regulation, as evidenced by both GATA-3 and ER association to *cis*-regulatory elements of this gene [(17), Figure 4], it was important to examine the contribution of KDM4B to GATA-3–mediated receptor expression independently of the chromatin-bound ER. Our data provide evidence that GATA-3 is a KDM4B-interacting protein, adding to the growing list of transcriptional regulators already shown to associate with this demethylase, including components of the SWI/SNF-B (15) and MLL2 (14) complexes. Furthermore, re-ChIP experiments (Figure 4C) and KDM4B depletion studies (Figure 5) indicate that the GATA-3–KDM4B interaction is evident on enhancer and promoter elements of the *ER* gene, in both basal and estrogen-stimulated conditions, and that KDM4B is required to reduce H3K9me3 levels at these loci permitting GATA-3 binding and subsequent up-regulation of receptor expression. Importantly, experiments conducted in the absence of estrogen or in the presence of Tamoxifen or the SERD fulvestrant demonstrated that KDM4B depletion effectively down-regulated basal ER expression, indicating that the enzyme was functioning via GATA-3, and independently of the receptor, to facilitate *ER* gene transcription (Supplementary Figures S6, S14 and S15). This concept is further supported by luciferase reporter experiments demonstrating KDM4B-mediated co-activation of GATA-3 transcriptional activity on the *ER* gene Enh2 promoter element (Supplementary Figure S13), although this was not evident on the Enh1 element, which may be a consequence of a non-physiological assay using *cis*-regulatory elements in isolation. Importantly, our data describe a novel role for KDM4B as a co-regulator of GATA-3 on the *ER* gene. Whether KDM4B functions as a global regulator of GATA-3 remains to be addressed, but given that GATA-3 acts as a pioneer factor for a subset of ER-target genes (30) may suggest that it plays an important role in controlling the chromatin deposition and/or function of the transcription factor within the receptor signalling cascade.

To further investigate the role of KDM4B in the ER signalling cascade, we addressed the potential role of the enzyme in controlling the function of the pioneer factor FOXA1. Deposition of FOXA1 into enhancer elements neighbouring ER-target genes is largely indispensable for estrogen-dependent transcription (25,31), but how this process is controlled remains ill-defined. ChIP-sequencing–based experiments have indicated FOXA1 enrichment at regions of chromatin enriched for H3K4me2 and depleted of H3K9me2 marks (31), suggesting a potential role for H3K9 demethylase enzymes in the potentiation of FOXA1–chromatin association. To this end, we undertook KDM4B-depletion studies to address if this impacted on focal and global association of the pioneer factor using respective ChIP and chromatin fractionation experiments. Unfortunately, these studies were hindered by the intriguing finding that FOXA1 expression was markedly reduced in response to KDM4B knock-down, indicating that although we are unable to examine the function of the enzyme in controlling FOXA1 chromatin deposition, we have identified an additional component of the ER signalling cascade that is transcriptionally regulated by KDM4B. FOXA1 has previously been shown to be directly up-regulated in MCF-7 cells by estrogen treatment, suggesting it is a direct ER-target gene (32). Therefore, reduced FOXA1 levels in response to KDM4B depletion (Figure 6) may simply be a consequence of down-regulated ER protein levels. However, given that this effect was evident in conditions where the ER was inactive does suggest that additional factors may be co-operating with KDM4B to regulate FOXA1 transcription. ChIP experiments demonstrated direct KDM4B association upstream of the *FOXA1* gene and depletion of the demethylase attenuated removal of repressive H3K9me3 marks required for estrogen-dependent FOXA1 expression (Supplementary Figure S19). Intriguingly, the three *cis*-regulatory elements upstream of the *FOXA1* gene that showed KDM4B association are enriched for GATA-3-binding sites, suggesting that interplay between these two proteins may, again, regulate transcription of an important regulator of the ER signalling cascade. To add further complexity to the interplay between KDM4B and components of the ER signalling system, we also showed that FOXA1 was recruited to the *ER* gene promoter and enhancers in response to hormone stimulation (Supplementary Figure S20), suggesting that the effect of KDM4B knock-down on *ER* expression may be a consequence of a combination of lower FOXA1 levels and attenuated recruitment of GATA-3 to upstream regulatory elements of the *ER* gene. Future studies using KDM4B overexpression model systems may provide a more suitable background for examining the role of enhancer demethylation by this enzyme to regulate FOXA1–chromatin association.

The development of therapeutic agents to target many of the characterized epigenetic enzymes, so-called ‘epi-inhibitors’, provides several opportunities for the treatment of a plethora of diseases including cancer. Given the role of several HDM enzymes in the propagation and progression of malignancy and neurological disorders (33), there is substantial interest in the development of demethylase inhibitors (HDMi) for the treatment of these diseases. Importantly, this work and findings from a number of translational studies (14,15,16) suggest KDM4B is a realistic therapeutic target for estrogen-dependent BCa treatment; KDM4B is overexpressed in aggressive disease, depletion of the enzyme reduces BCa cell growth *in vitro* and *in vivo* and enhances BCa cell cytotoxicity (Supplementary Figure S11) without affecting growth of ER negative cell lines (14,15). Although at present there are no indications of recurrent KDM4B mutations in BCa, or indeed other cancer types, acquisition of activating mutations in KDM4B or *KDM4B* gene silencing by methylation may be a contributory factor in the persistence or loss of ER expression during BCa development and response to treatment. This is an important avenue that needs further exploration. Importantly, KDM4B knockout animals are phenotypically normal (15), indicating that the enzyme is not a general regulator of cellular proliferation and aberrant expression in BCa is acquired on cellular transformation, suggesting targeting KDM4B is potentially tumour specific. KDM4 family members, including KDM4A-E, demonstrate >98% sequence identity within the catalytic JmjC domain, highlighting selective targeting of the enzyme may not be achievable. However, the recent demonstration that KDM4C is a target gene and co-regulator of hypoxia-inducible factor 1, and demonstrates elevated expression in invasive BCa and is required for lung metastasis *in vivo* (34), suggests that a pan-KDM4 family inhibitor may be beneficial to patients who have failed neo-adjuvant therapy but retain expression of the ER.

In summary, our data demonstrate that KDM4B is a master regulator of the ER signalling cascade by controlling ER and FOXA1 expression and that these gene targets are likely to be a major contributing factor to the KDM4B-knock-down phenotype seen in BCa cell lines *in vitro* and *in vivo* (14,15). Ultimately, are findings support the development of agents to down-regulate KDM4B activity for BCa treatment and have identified, in the form of the ER and FOXA1, two potential biomarkers for drug development programmes.

## SUPPLEMENTARY DATA

Supplementary Data are available at NAR Online: Supplementary Tables 1 and 2 and Supplementary Figures 1–20.

## FUNDING

Cancer Research UK (to J.S., K.C., D.O. and J.W.); Association for International Cancer Research (to M.W.); Breast Cancer Campaign (L.G.). Funding for open access charge: Newcastle University.

*Conflict of interest statement*. None declared.

## Supplementary Material

Supplementary Data
