# Supplementary material for: KDM4B is a Master Regulator of the Estrogen Receptor Signalling Cascade
Source: Nucleic Acids Res. 2013 May 30;41(14):6892–904. doi: 10.1093/nar/gkt469 (PMC3737554; doi:10.1093/nar/gkt469)
Supplement: Supplementary Data [file supp_41_14_6892__index.html]

KDM4B is a Master Regulator of the Estrogen Receptor Signalling Cascade — KDM4B is a Master Regulator of the Estrogen Receptor Signalling Cascade — Supplementary Data 

# KDM4B is a Master Regulator of the Estrogen Receptor Signalling Cascade

## Supplementary Data

files

**Files in this Data Supplement:**

- Supplementary Data - pdf file
