# Supplementary material for: KDM4B is a Master Regulator of the Estrogen Receptor Signalling Cascade
Source: Nucleic Acids Res. 2013 May 30;41(14):6892–904. doi: 10.1093/nar/gkt469 (PMC3737554; doi:10.1093/nar/gkt469)
Supplement: Supplementary Data [file supp_gkt469_nar-03276-x-2012-File008.pdf]

Supplementary Information

To accompany Gaughan *et al.*, '<u>KDM4B is a Master Regulator of the ER Signalling Cascade</u>'

<u>Figure Legends</u>

Supplementary Figure S1. KDM4B regulates ER transcriptional activity. A. MCF-7 cells were transiently transfected with either scrambled (Scr) control or KDM4B (siKDM4B) siRNAs in steroid-depleted media and treated with 10 nM E_2 for up to 48 hours before RNA extraction. Resultant cDNA was incorporated into quantitative PCR analysis to assess pS2 and progesterone receptor (PgR) mRNA expression. Data is the mean of three independent experiments +/- standard error. B. Individual or pooled siKDM4B siRNAs ((A),(B),(C))(P) and pS2 (sipS2) and ER (siER) targeting siRNAs were transiently transfected into MCF-7 cells grown in serum-containing media (upper panel) or steroid-depleted media supplemented with and without 10 nM E_2 (lower panel). KDM4B and pS2 expression was assessed as described above.

Supplementary Figure S2. ER association and H3K9me3 levels demonstrate estrogen-dependent flux upon *pS2* and *GREB1* promoters. MCF-7 cells were grown in steroid-depleted media for 48 hours prior to treatment with or without 10 nM E_2 for 45 and 180 minutes and then subject to ChIP using anti-ER and –H3K9me3 antibodies. Data represents the average of three independent experiments +/- standard error.

Supplementary Figure S3. KDM4B depletion reduces H3K9Ac at the *pS2* promoter. MCF-7 cells were transiently transfected with either scrambled (Scr) control or KDM4B siRNA (siKDM4B) in steroid-depleted media for 48 hours with or without 45 minute E_2 stimulation prior to ChIP with an anti-H3K9Ac antibody. Resultant DNA was analysed by quantitative PCR using primers specific to the *pS2* estrogen response element (ERE). ChIP data is an average of three independent experiments +/- standard error.

Supplementary Figure S4. KDM4B depletion impacts on global histone methylation and acetylation levels. MCF-7 cells grown in serum-containing media were transfected with either scrambled (Scr) or KDM4B (siKDM4B) siRNAs for 48 hours prior to acid extraction of histone proteins and Western analysis using antibodies to H3K9me1, H3K9me2, H3K9me3, H3K9Ac, histone H3 and KDM4B.

Supplementary Figure S5. KDM4B depletion reduces ER expression in estrogen-independent MCF-7 cells. Estrogen-independent MCF-7 (EI-MCF-7) cells were transiently transfected with either scrambled (Scr) control or KDM4B (siKDM4B) siRNAs for 40 hours prior to treatment with 10 nM E_2 for 8 hours. Cells were lysed in SDS-sample buffer and subject to Western analysis using anti-KDM4B, ER and α-Tubulin antibodies.

Supplementary Figure S6. Basal ER expression is reduced upon KDM4B depletion. MCF-7 cells were transiently transfected with either scrambled (Scr) control or KDM4B (siKDM4B) siRNAs in steroid-depleted media for 40 hours prior to 10 nM E_2 treatment for up to 8 hours before RNA extraction. Resultant cDNA was incorporated into quantitative PCR to assess pS2 and ER mRNA levels. Data is the mean of three independent experiments +/- standard error.

Supplementary Figure S7. ER expressing cell lines T47D, MCF-7 and EI-MCF-7 display variable KDM4B expression levels. Lysates of MCF-7 and T47D cells grown in serum-containing media and EI-MCF-7 cells grown in steroid-depleted media were subject to SDS-PAGE and immunoblotted with KDM4B and α-Tubulin antibodies.

Supplementary Figure S8. Ectopic expression of KDM4B does not affect ER stability. MCF-7 cells grown in serum-containing media were transiently transfected with either wild-type KDM4B, demethylase-dead $KDM4B_{H189G/E191Q}$ mutant or empty mammalian expression vectors for 40 hours prior to treatment with 1 µM cycloheximide (CHX) for up to 8 hours. Samples were subsequently analysed by immunoblotting using antibodies to KDM4B, ER and α-Tubulin.

Supplementary Figure S9. KDM4B depletion reduces ER expression in MCF-7 and BT-474 BCa cell lines. MCF-7 (A) and BT-474 (B) cells were transiently transfected with either scrambled (Scr) control or KDM4B (A)-(C) siRNAs (siKDM4B (A-C) for 48 hours prior to RNA extraction and quantitative PCR analysis to assess pS2 and ER expression levels, or Western analysis using antibodies to KDM4B, ER and α-Tubulin. Quantitative PCR data is a mean of three independent experiments +/- standard error.

Supplementary Figure S10. Additional KDM4B siRNAs reduce ER expression in BCa cells. A. MCF-7 cells were transiently transfected with either scrambled (Scr) control or two KDM4B siRNAs (siKDM4B (C) or (D)) in steroid-depleted media for 40 hours prior to treatment with 10 nM E_2 for 8 hours. Cells were harvested for quantitative PCR analysis to assess pS2 and GREB1 mRNA expression, or Western analysis using antibodies to ER, KDM4B and α-Tubulin. Quantitative PCR data is a mean of three independent experiments +/- standard error. B. MCF-7 and T47D cells grown in serum-containing media were transiently transfected as in (A) and ER, KDM4B and α-Tubulin levels analysed by immunoblotting. Duplicate samples (labelled (1) and (2)) were analysed for MCF-7 cells.

Supplementary Figure S11. KDM4B depletion affects BCa cell proliferation and apoptosis. MCF-7 cells grown in steroid-depleted media supplemented with and without 10 nM E_2 and T47D cells grown in serum-containing media were transiently transfected with either scrambled control (Scr) or two KDM4B siRNAs (siKDM4B (C) or (D)) and after 96 hours WST1 proliferation (A) or BrdU incorporation assays (B) were performed. Data is the mean of three independent experiments +/- standard error (* $p<0.05$, Student T-test).

Supplementary Figure S12. GATA-3 depletion reduces ER expression. MCF-7 (A) and T47D (B) cells were transiently transfected with either scrambled (Scr) control or two GATA-3 siRNAs (siGATA-3 (1), siGATA-3 (2)) in steroid-depleted media for 40 hours prior to treatment with 10 nM E2 for 8 hours and subject to Western analysis using antibodies to ER, KDM4B, GATA-3 and α-Tubulin. A short and long exposure for GATA-3 immunoblotting is shown.

Supplementary Figure S13. KDM4B co-activates GATA-3 upon the *ER Enh2* luciferase reporter in MCF-7 and HEK293T cells. MCF-7 cells (A) or HEK293T cells (B) grown in steroid-depleted media were transiently transfected with or without GATA-3 (50 ng/well) and increasing amounts of KDM4B (10-250ng/well) mammalian expression vectors together with either Enh1 (left panels) or Enh2 (right panels) and β-galactosidase reporter constructs. After 48 hours, cells were harvested for luciferase and β-galactosidase activities and data is representative of N=3 experiments +/- standard error (*denotes $p<0.05$).

Supplementary Figure S14. KDM4B controls *ER* gene expression independently of the ER. MCF-7 (A) and T47D (B) cells were transiently transfected with either scrambled (Scr) control, two KDM4B siRNAs (siKDM4B (C) and siKDM4B (D)), or an ER oligonucleotide (siER) as a positive control, in steroid-depleted media for 40 hours prior to treatment with 10 nM E_2, supplemented with and without 1 µM 4-hydroxy-Tamoxifen (Tam), for 8 hours followed by RNA extraction. Resultant cDNA was incorporated into quantitative PCR analysis to examine pS2 and ER expression. Data is the mean of three independent experiments +/- standard error.

Supplementary Figure S15. Fulvestrant treatment does not affect KDM4B recruitment to *ER* gene *cis*-regulatory elements. A. MCF-7 cells were grown in steroid-depleted media for 48 hours prior to vehicle or 1 µM fulvestrant pre-treatment for 195 minutes and then treated with or without 10 nM E_2 for an additional 45 minutes before ChIP using an anti-ER antibody or isotype control IgG. Recruitment to ER target genes *pS2* and *GREB1* was analysed by quantitative PCR. Data is an average of three independent experiments +/- standard error. B. Cells treated as above were subject to Western analysis using ER, KDM4B and α-Tubulin antibodies. C. MCF-7 cells grown in steroid-depleted media for 48 hours were treated as in (A) with vehicle or 1µM fulvestrant for 195 minutes and then stimulated with 10 nM E_2 for 45 minutes prior to ChIP using an anti-KDM4B antibody or isotype control. KDM4B enrichment is shown at Pro A, Enh1 and Enh2 regions of the ER gene. Data is the mean of three independent experiments +/- standard error.

Supplementary Figure S16. EI-MCF-7 cells show altered requirement for KDM4B at *ER* gene *cis*-regulatory elements. EI-MCF-7 cells grown in steroid-depleted media were transiently transfected with either scrambled (Scr) or KDM4B siRNAs for 72 hours prior to treatment with 10 nM E_2 for 45 minutes and subsequent ChIP analysis using an antiH3K9me3 antibody or isotype control. H3K9me3 enrichment is shown at Pro A, Enh1 and Enh2 regions of the *ER* gene. Data is the mean of three independent experiments +/- standard error.

Supplementary Figure S17. KDM4B depletion does not affect cellular GATA-3 levels. MCF-7 (A) and T47D (B) cells were transiently transfected with either scrambled (Scr) control or KDM4B (siKDM4B) siRNAs in steroid-depleted media for 40 hours prior to treatment with 10 nM E_2 for 8 hours and Western analysis using anti-GATA-3, KDM4B and α-Tubulin antibodies.

Supplementary Figure S18. Ectopic KDM4B expression does not affect FOXA1 stability. MCF-7 cells were transiently transfected with a KDM4B mammalian expression vector, or an empty vector control, for 40 hours prior to treatment with 1 μM cycloheximide (CHX) for up to 8 hours prior to Western analysis using anti-KDM4B, FOXA1 and α-Tubulin antibodies.

Supplementary Figure S19. KDM4B is recruited to and regulates H3K9me3 levels at FOXA1 promoter elements. A. Diagrammatic representation of the position of the upstream regions of the FOXA1 gene tested for KDM4B association including sites 1, 2 and 5 kb upstream from the transcriptional start-site (+1). B. MCF-7 cells were grown in steroid-depleted media for 48 hours prior to treatment with or without 10 nM E_2 for 45 or 180 minutes before ChIP utilising an anti-KDM4B antibody or isotype control. C. MCF-7 cells grown in steroid-depleted media were transfected with either scrambled (Scr) or KDM4B siRNAs for 72 hours prior to 10 nM E_2 treatment for 45 minutes and ChIP using an anti-H3K9me3 antibody or isotype control. KDM4B and H3K9me3 enrichment was assessed at FOXA1 1, 2 and 5 kb regions using quantitative PCR and data represents the average of three independent experiments +/- standard error.

Supplementary Figure S20. FOXA1 is recruited to ER gene promoter and enhancer elements in response to estrogen. MCF-7 cells grown in steroid-depleted media for 48 hours were treated with and without 10 nM E_2 for 45 or 180 minutes before ChIP using an anti-FOXA1 antibody or isotype control. Enrichment of FOXA1 was assessed at *ER* gene promoter and enhancer elements Pro A, Enh1 and Enh2 by quantitative PCR. Data represents the average of three independent experiments +/- standard error.

Supplementary Figure S1

A.

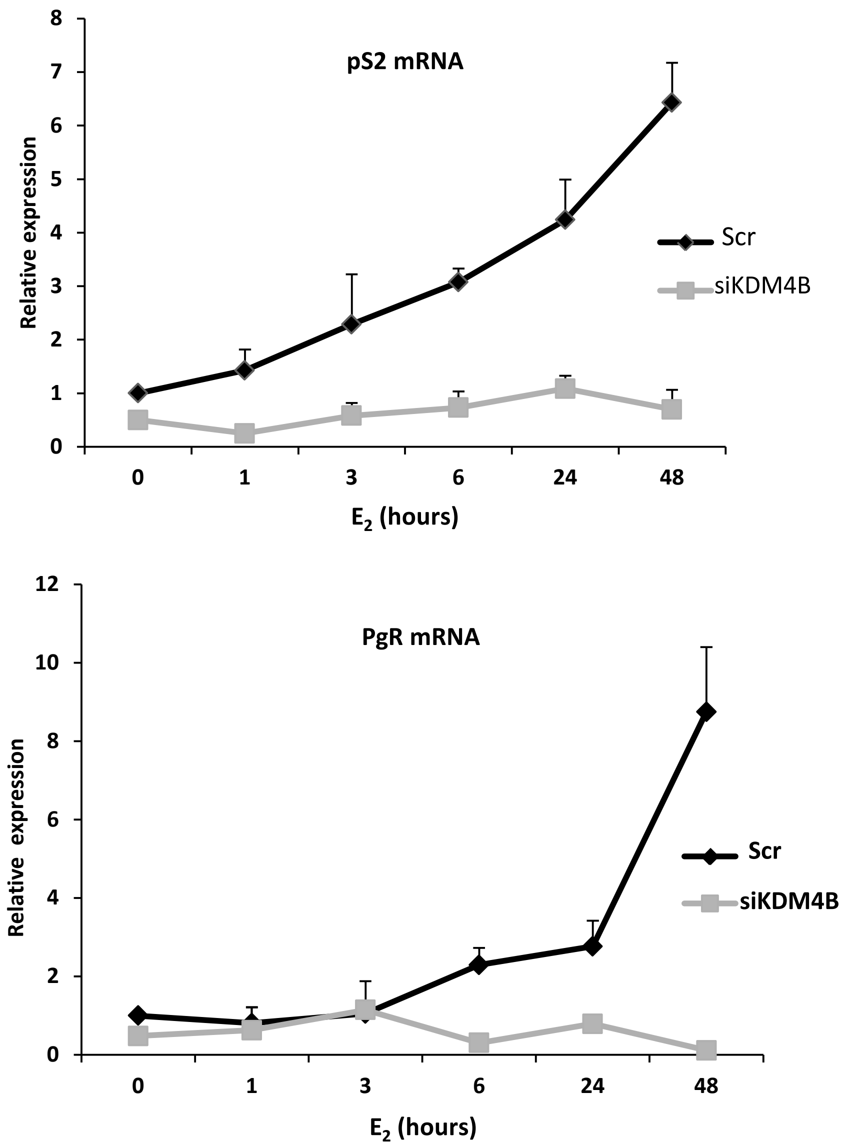

B.

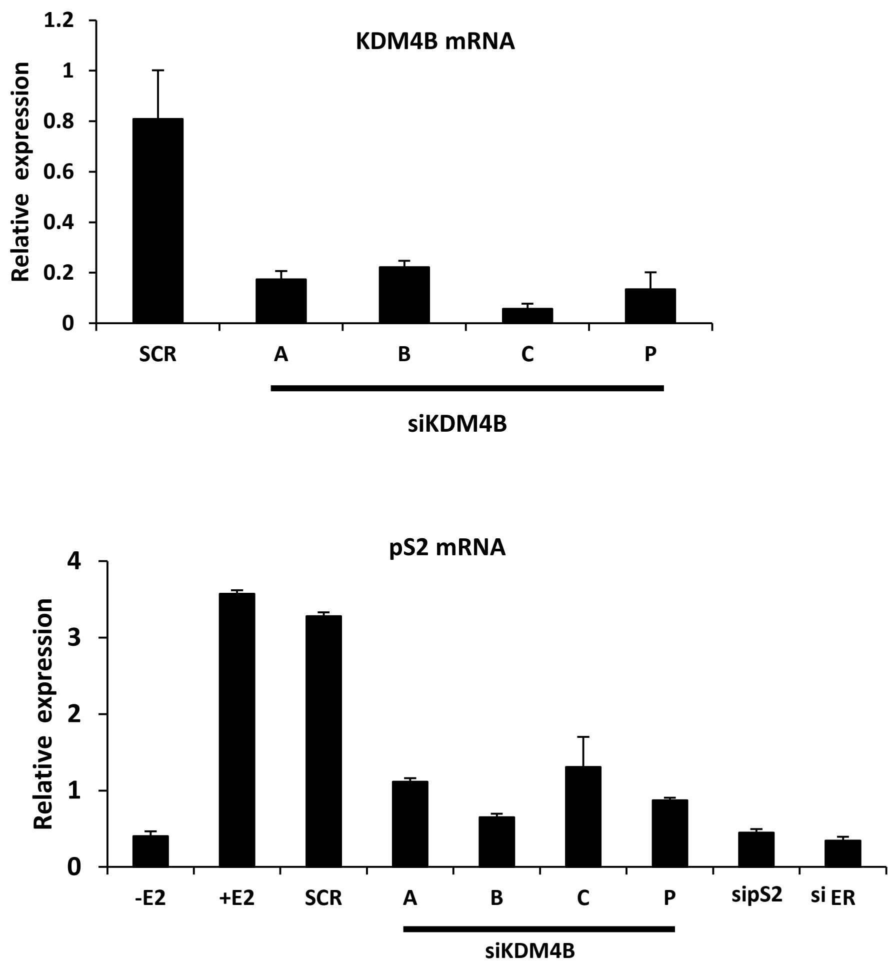

Supplementary Figure S2

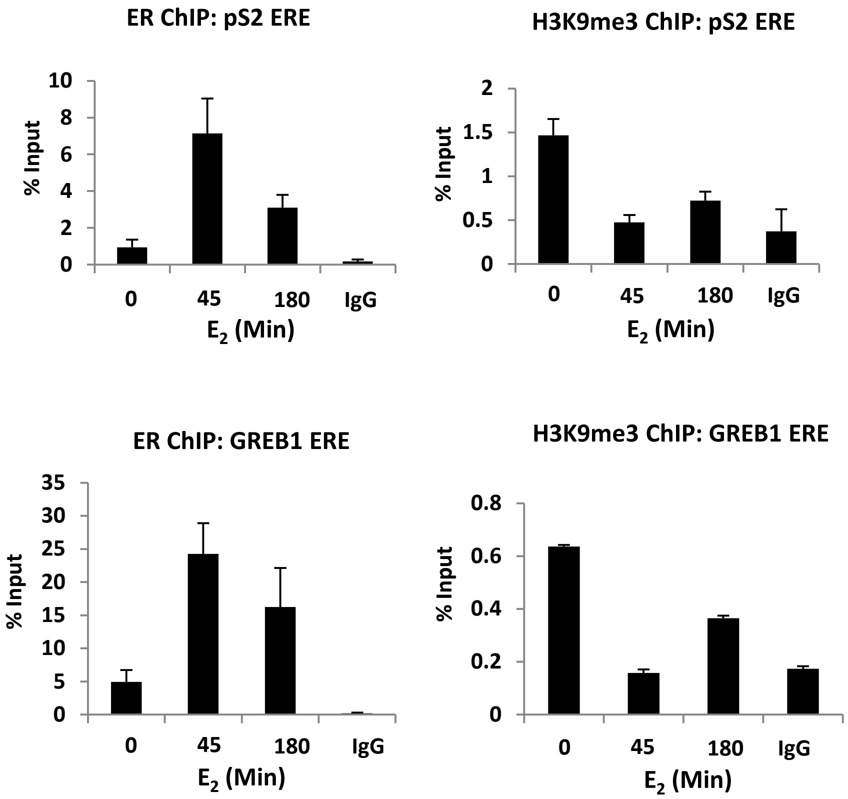

Supplementary Figure S3

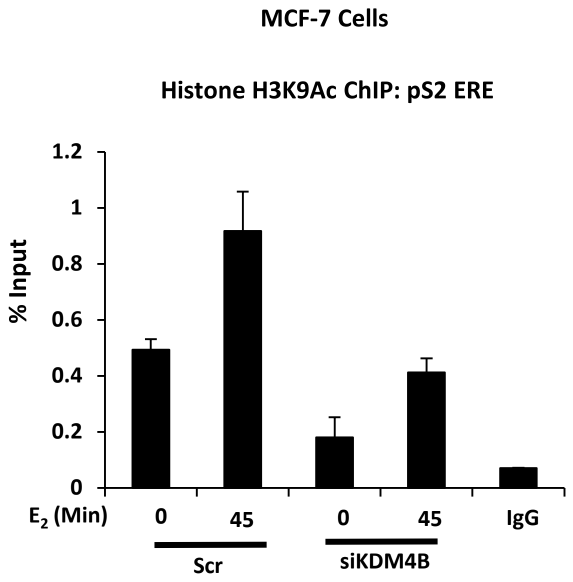

Supplementary Figure S4

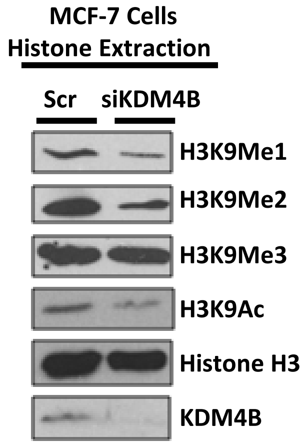

Supplementary Figure S5

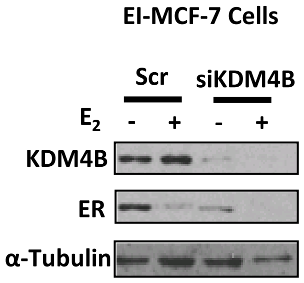

Supplementary Figure S6

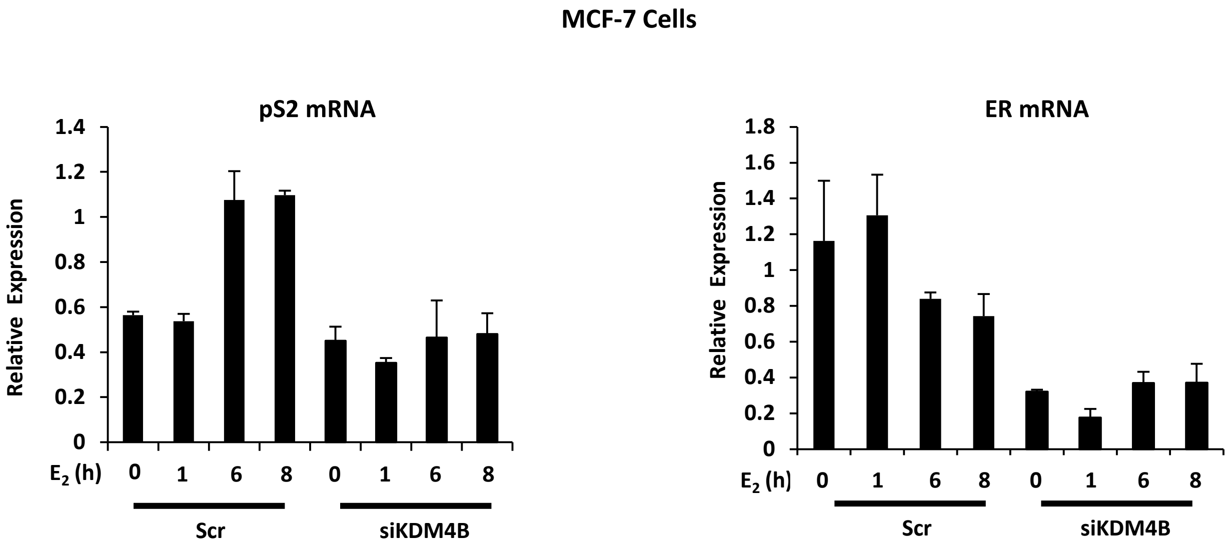

Supplementary Figure S7

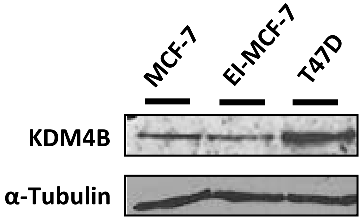

Supplementary Figure S8

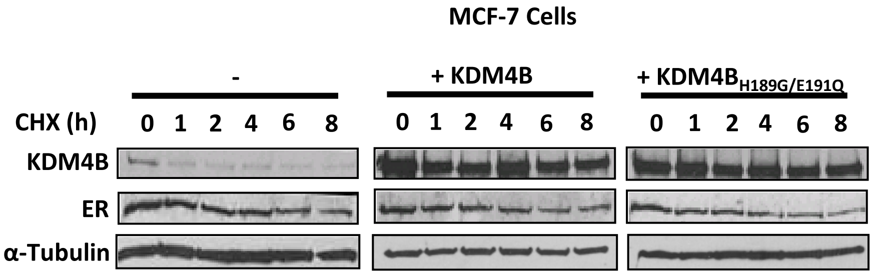

Supplementary Figure S9

MCF-7 Cells

A.

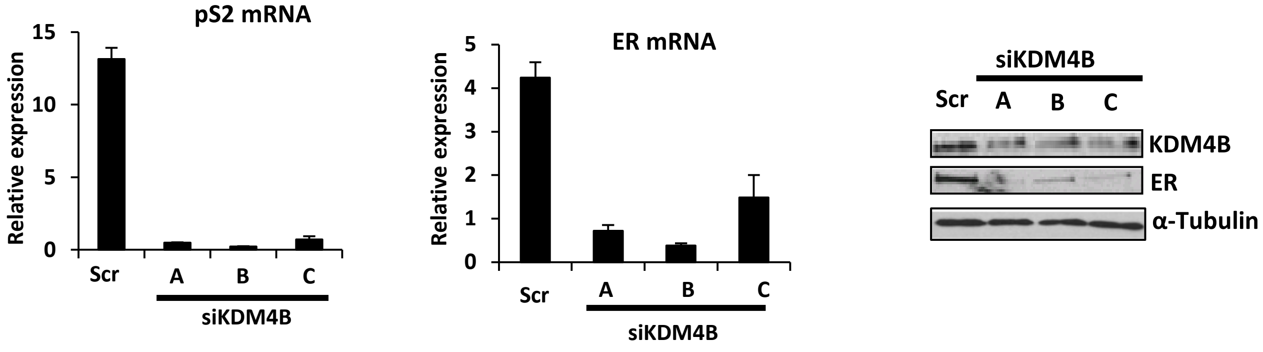

BT-474 Cells

B.

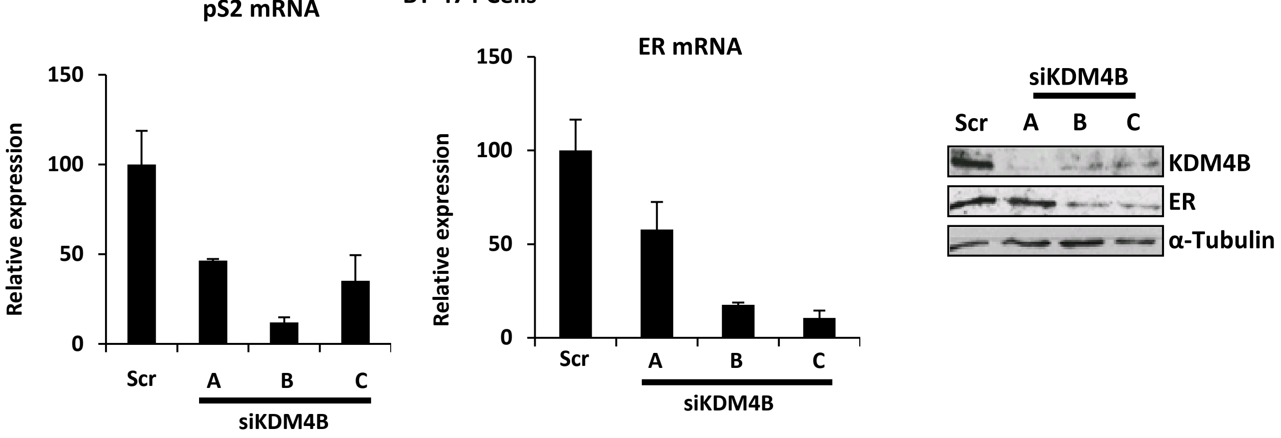

Supplementary Figure S10

A.

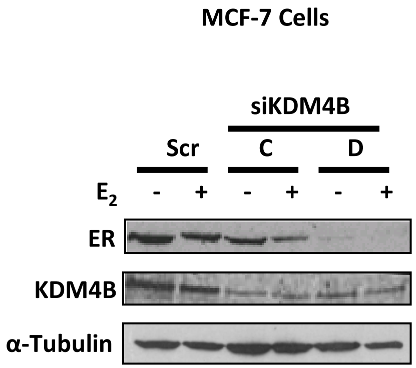

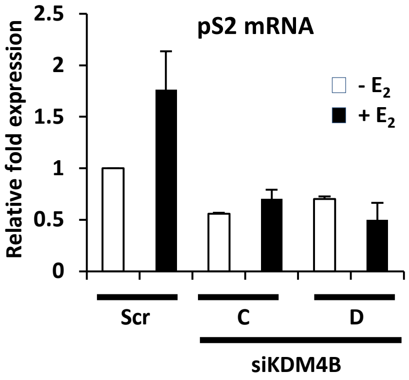

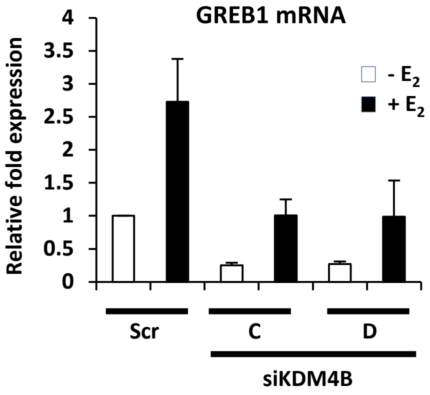

B.

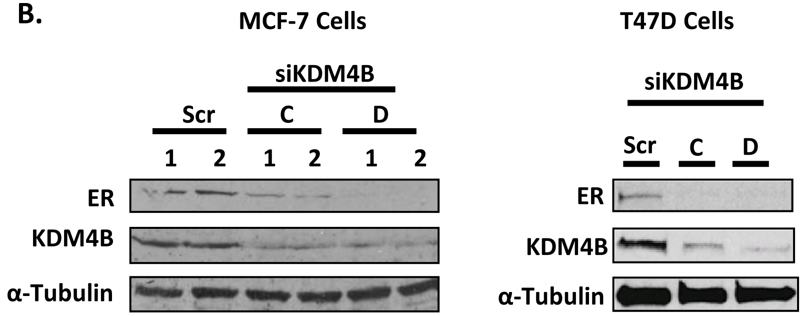

Supplementary Figure S11

A.

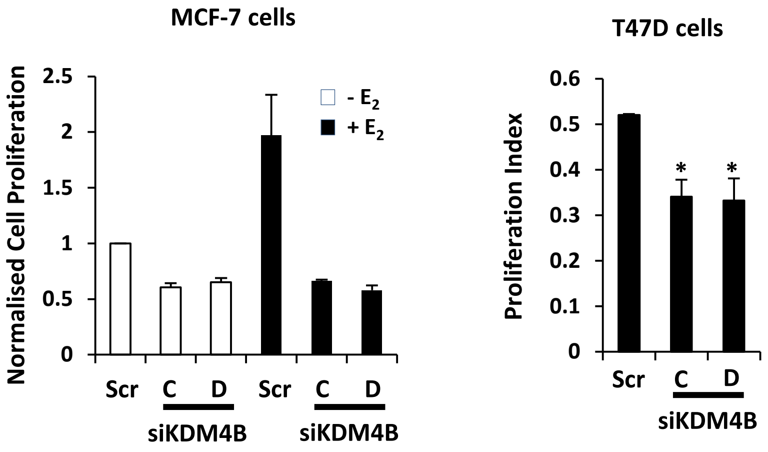

B.

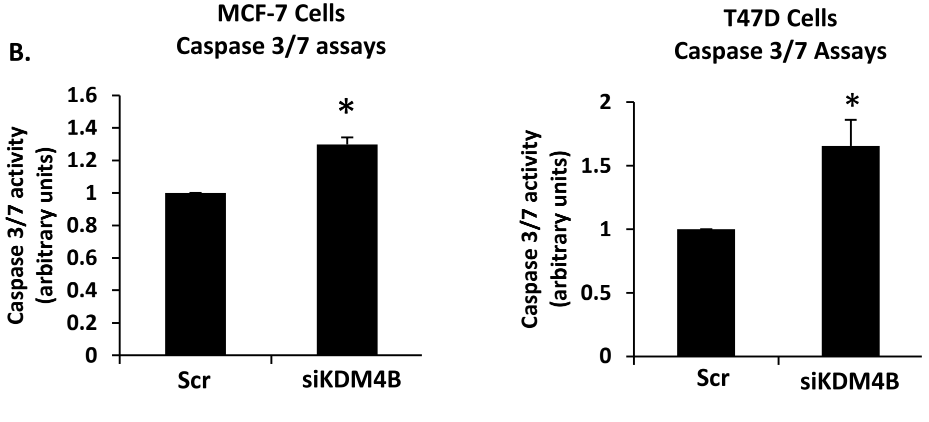

Supplementary Figure S12

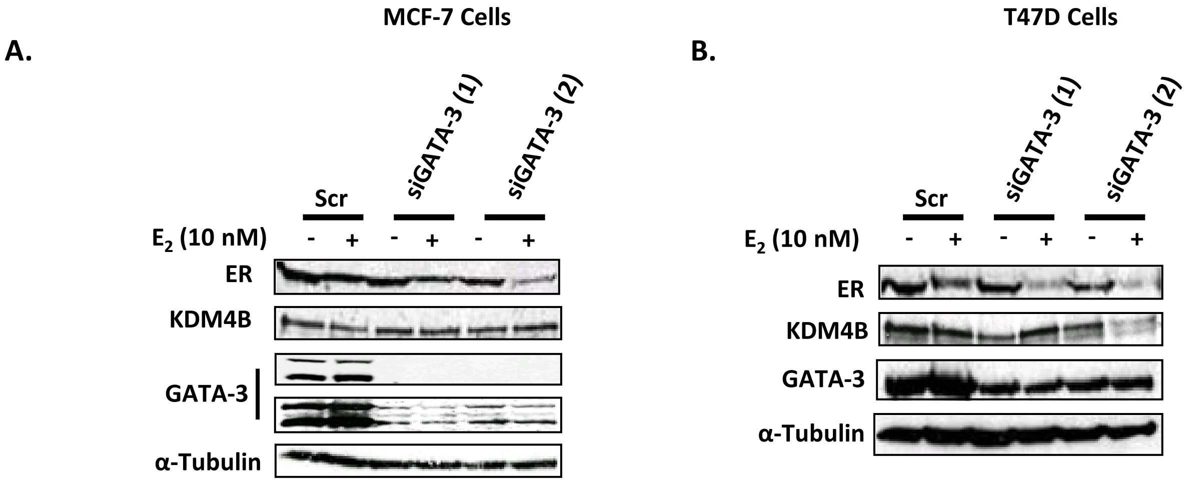

Supplementary Figure S13

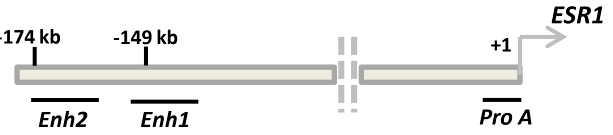

A.

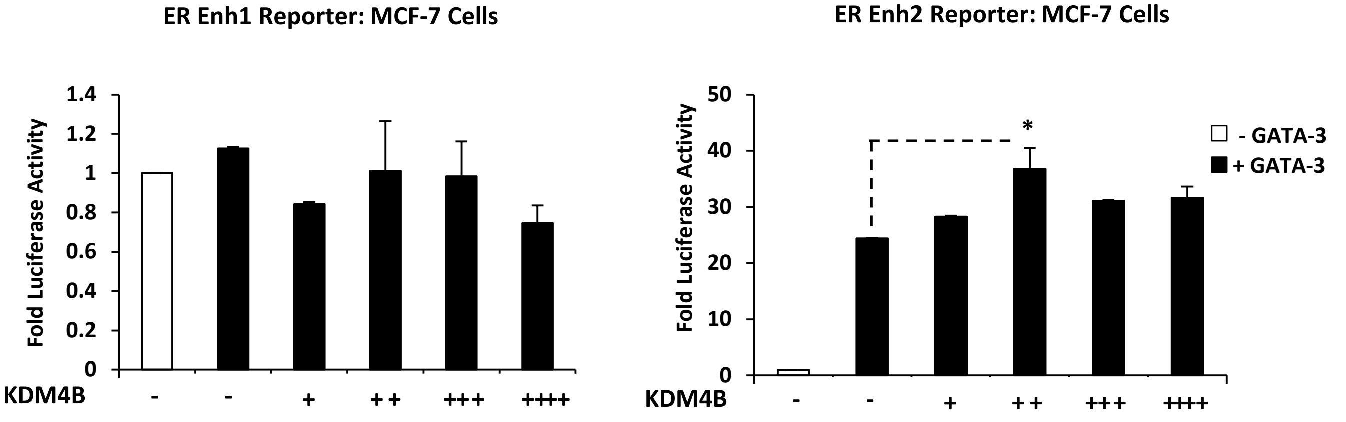

B.

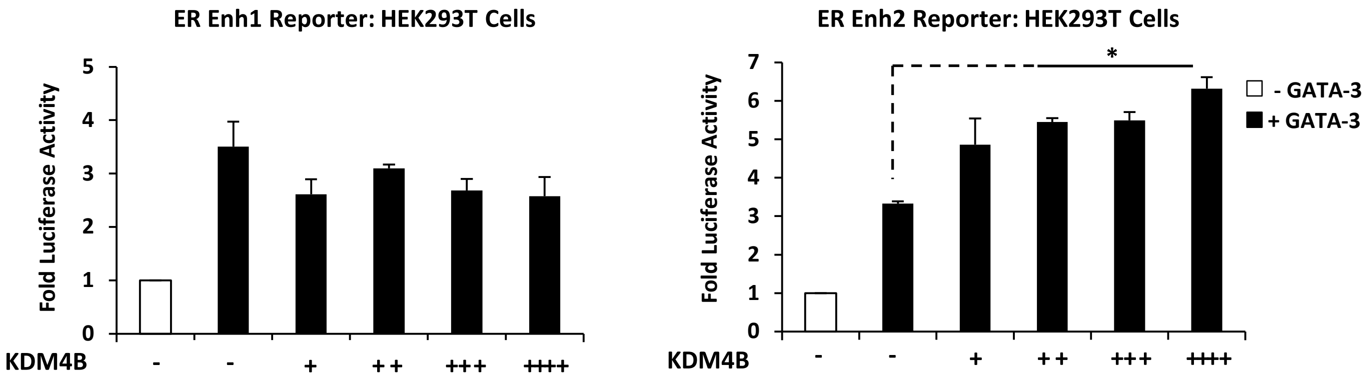

Supplementary Figure S14

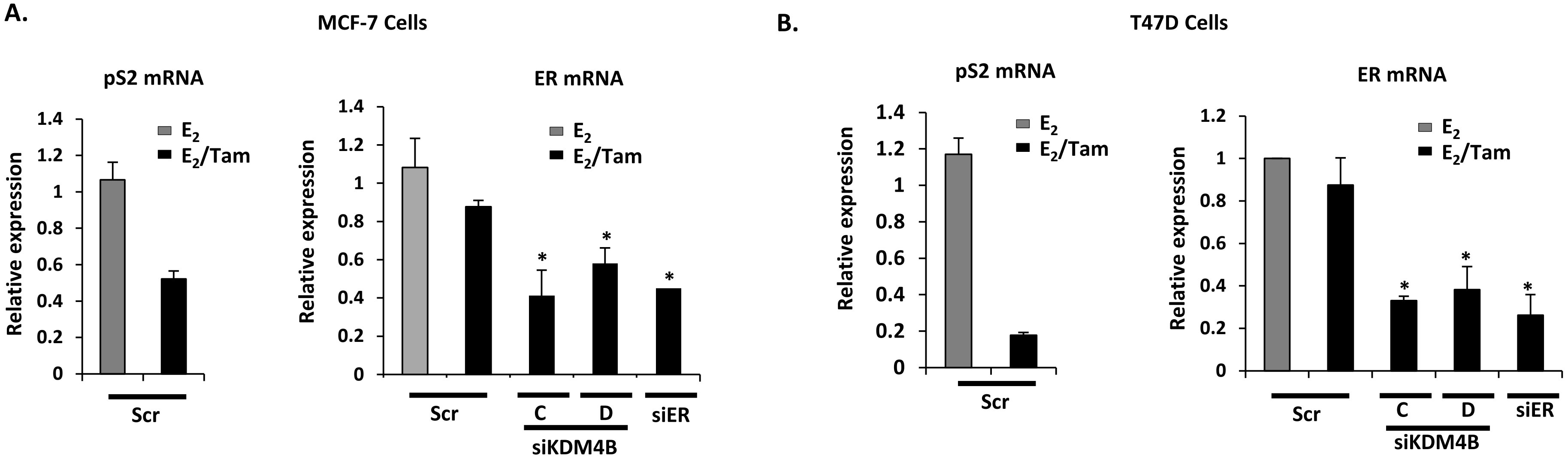

Supplementary Figure S15

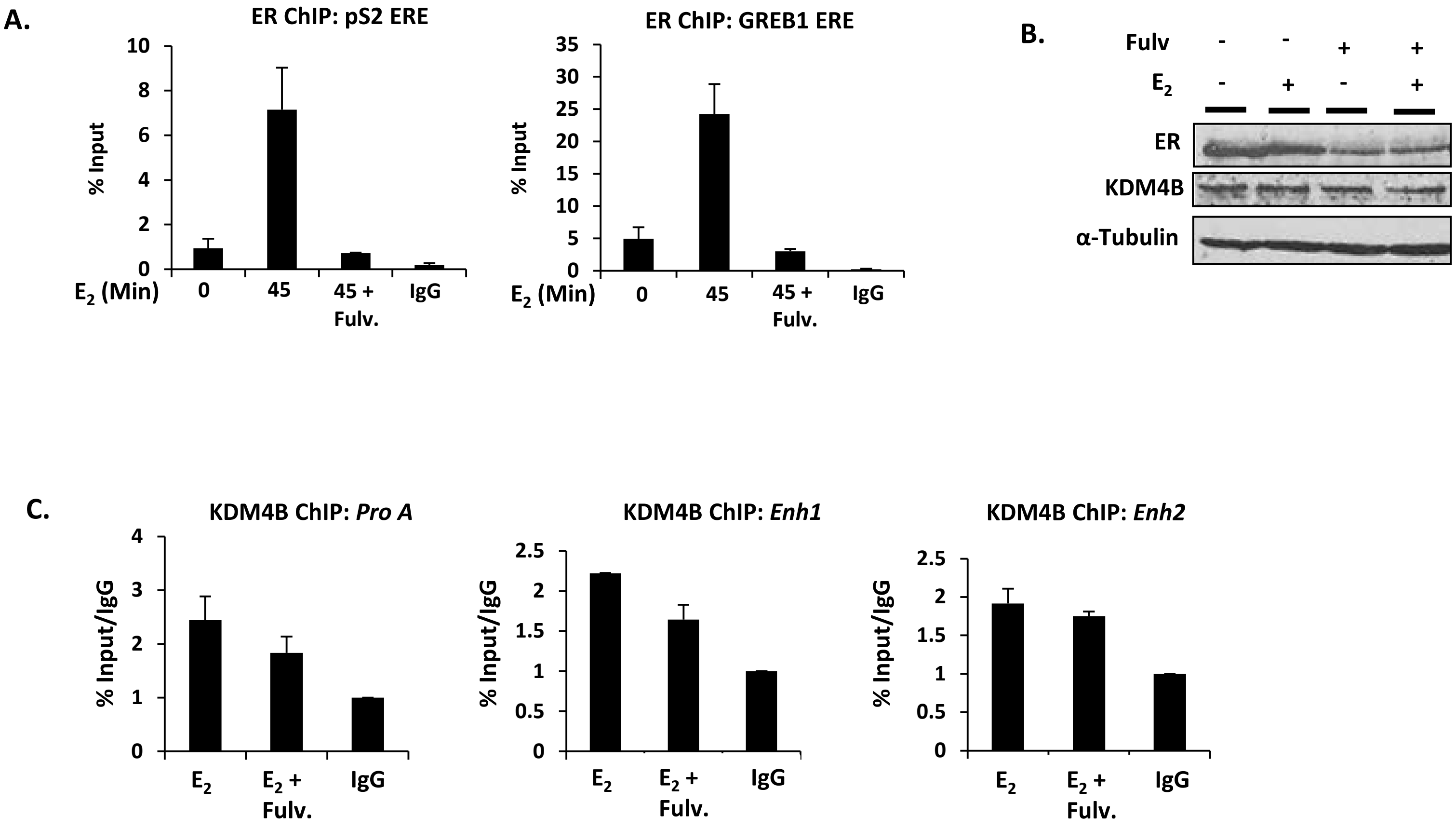

Supplementary Figure S16

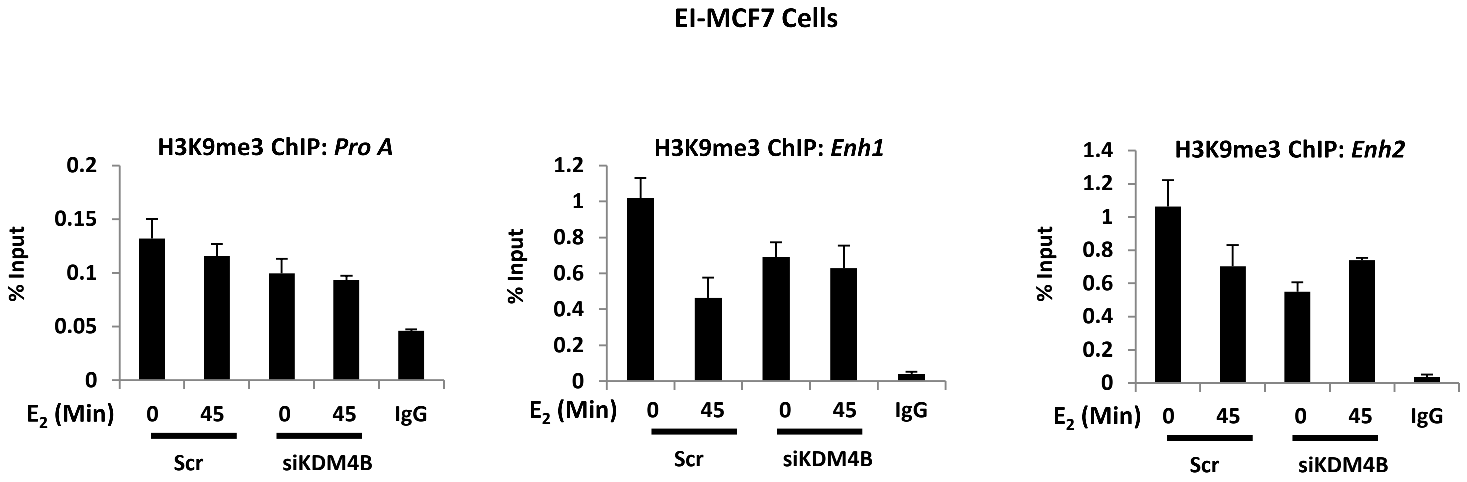

Supplementary Figure S17

A.

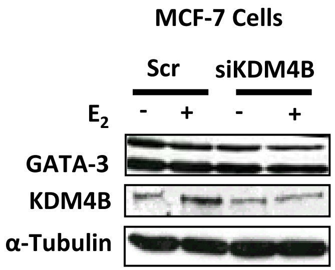

B.

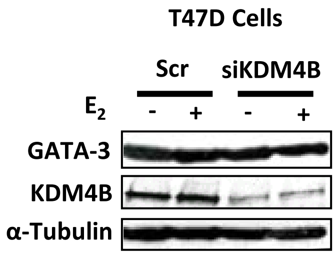

Supplementary Figure S18

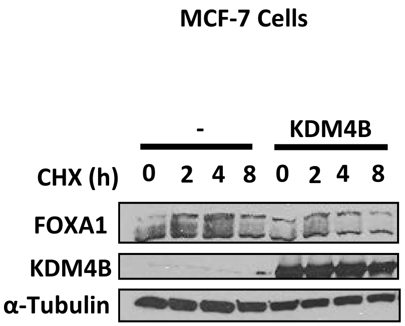

Supplementary Figure S19

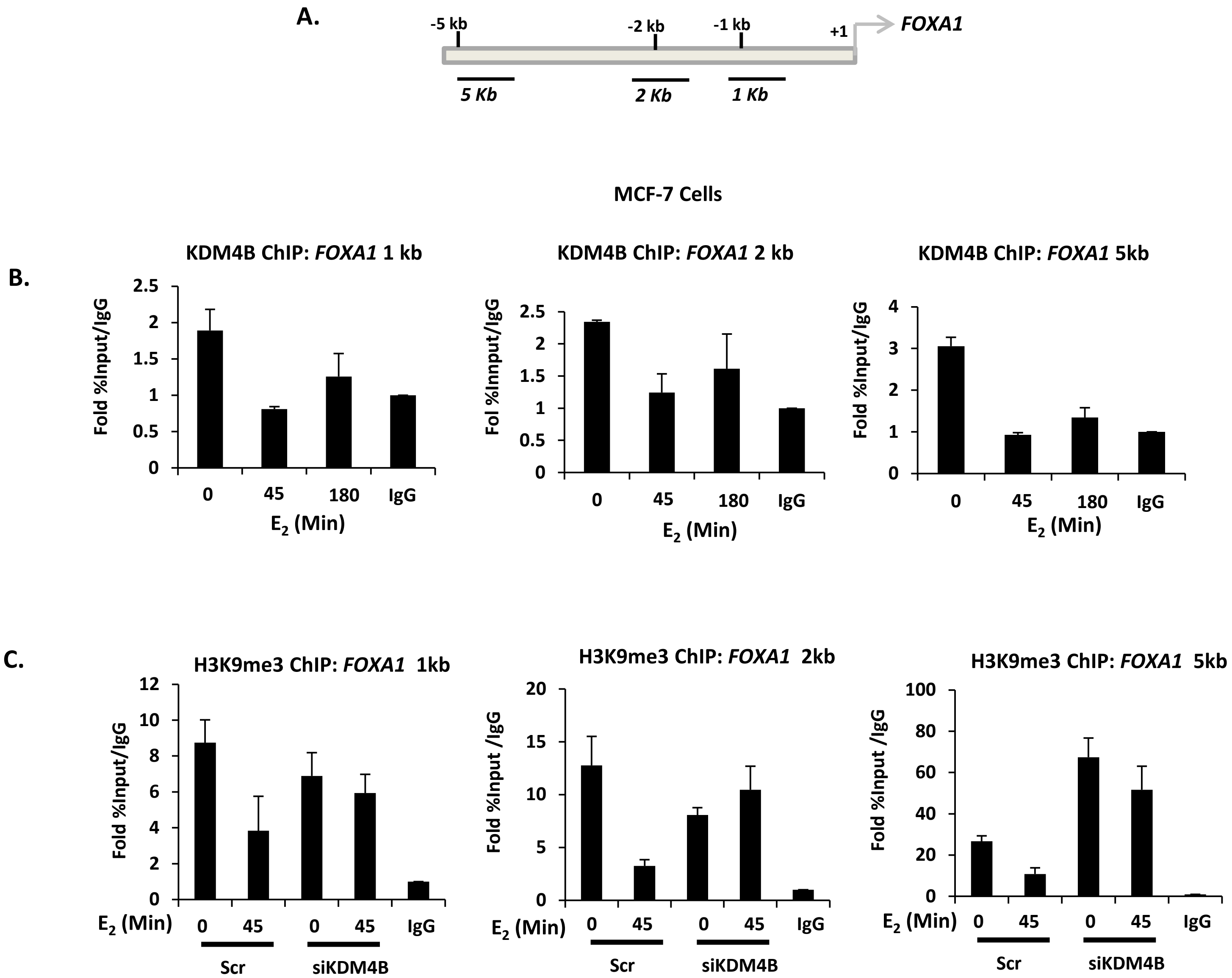

Supplementary Figure S20

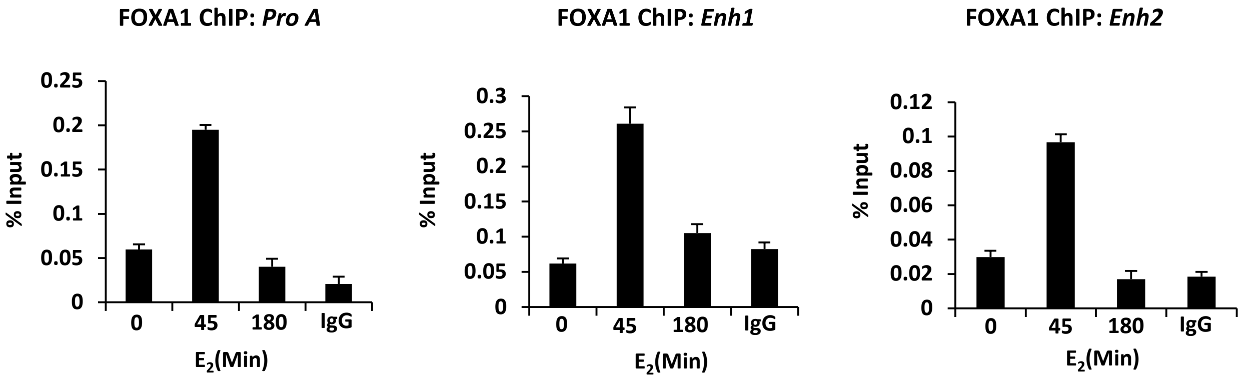

Supplementary Table S1

**ChIP Primers**	
Primer Name	Sequence (5'-3')
ER gene Enhancer 1 F<br>ER gene Enhancer 1 R	TGTAGGCTAGTTTTGTTTAACGATTTTT<br>GGTGATGGGAGAATTGCTTAGAA
ER gene Enhancer 2 F<br>ER gene Enhancer 2 R	GCTCTTAAGGGTTCCTGGTGG<br>CAGTCTTGGCCCTTTACCCA
ER gene Promoter A F<br>ER gene Promoter A R	CACATAAGGCAGCACATTAGAGAAA<br>GAAGACTGGGCTTAAAATAAACGC
pS2 Promoter F<br>pS2 Promoter R	CACCCCGTGAGCCACTGT<br>CTGCAGAAGTGATTCATAGTGAGAGAT
GREB1 Promoter F<br>GREB1 Promoter R	AGCAGTGAAAAAAAGTGTGGCAACTGGG<br>CGACCCACAGAAATGAAAAGGCAGCAAACT
**mRNA Primers**	
pS2 F<br>pS2 R	GTGTCACGCCCTCCCAGT<br>GGACCCCACGAACGGTG
GREB1 F<br>GREB1 R	CAGACCACCACAACCACACTCT<br>GGATGCCTTCCTTCTTCATAGTCA
PgR F<br>PgR R	AGCCAGAGCCCACAATACAG<br>GACCTTACAGCTCCCACAGG
ER F<br>ER R	ACAAGCGCCAGAGAGATGAT<br>AAGGTTGGCAGCTCTCATGT
KDM4B F<br>KDM4B R	CATCTACCAGGTGGAGTTTGA<br>CTTCTGGGTGCTCAGCTCTT

**pGL3 Cloning Primers**	
Primer Name	Sequence (5'-3')
Enhancer 1 F<br>Enhancer 1 R	GCGCTCGAGAGGCTAGTTTTGTTTAATGA<br>CCGAAGCTTTTTAGAATAGAGTTTGGTGC
Enhancer 2 F<br>Enhancer 2 R	GCGCTCGAGATCCCAGATGGCCACCTGTT<br>CCGAAGCTTTCTGTCCTTGAGACTCATAA
**FOXA1 ChIP Primers**	
FOXA1 1 kb	TCGCTACAGATGACAAGGGG<br>GTGAGTCACAGCACCGGTTT
FOXA1 2 kb	TGATGGGACAACTTGCAGTCA<br>ACCTGGAACTCAGTAGGGCA
FOXA1 5 kb	CCTCTTTGTGTGAAGCGTGC<br>GGGCTTTCCCGCTCTACTTT

Supplementary Table S2

siRNA Name	Sequence (5’-3’)
KDM4B (A)	CTCTTCACGCAGTACAATA
KDM4B (B)	CAAATACGTGGCCTACATA
KDM4B (C)	GGCATAAGATGACCCTCAT
KDM4B (D)	GACCTGTACAGCATCAACT
ER	GGCAUGGAGCAUCUCUACA
GATA-3 (1)	AAGCCUAAACGCGAUGGAUAU
GATA-3 (2)	AACAUCGACGGUCAAGGCAAC
